# Gut microbiota modulation by Traditional Chinese Medicine: a translational strategy for metabolic dysfunction-associated steatotic liver disease

**DOI:** 10.3389/fphar.2025.1600439

**Published:** 2025-06-10

**Authors:** Ting Zhou, Ziwen Jin, Rilei Jiang, Weiwei Li

**Affiliations:** ^1^ School of Traditional Chinese Medicine, Shanghai University of Traditional Chinese Medicine, Shanghai, China; ^2^ Yueyang Hospital of Integrated Traditional Chinese and Western Medicine, Shanghai University of Traditional Chinese Medicine, Shanghai, China; ^3^ Longhua Hospital, Shanghai University of Traditional Chinese Medicine, Shanghai, China

**Keywords:** metabolic dysfunction-associated steatotic liver disease, gut microbiota, Traditional Chinese Medicine, gut-liver axis, bile acid metabolism

## Abstract

**Background:**

Metabolic dysfunction-associated steatotic liver disease (MASLD) has emerged as a critical global health burden, driven by rising prevalence rates and earlier disease onset. Current therapeutic strategies remain limited to lifestyle interventions, with no approved pharmacotherapies targeting disease progression. Growing evidence highlights gut microbiota dysbiosis as a pivotal contributor to MASLD pathogenesis, characterized by disrupted intestinal barrier function, endotoxin translocation, and dysregulated bile acid (BA) and short-chain fatty acid (SCFA) metabolism. Preclinical studies suggest that specific botanical drugs and standardized polyherbal formulations may mitigate MASLD through microbiota modulation.

**Methods:**

A systematic review of preclinical and clinical studies (2015–2025) was conducted across PubMed, Web of Science, and CNKI. Search terms included “gut microbiota,” “Traditional Chinese Medicine (TCM),” and “MASLD,” focusing on studies with chemically defined botanical metabolites (purity >90%) or rigorously characterized polyherbal formulations. Exclusion criteria eliminated reports lacking microbial taxonomic validation (e.g., 16S rRNA sequencing), dose-response relationships, or mechanistic validation in animal models.

**Results:**

The synthesis of studies reveals that TCM ameliorates MASLD through three interconnected mechanisms: restoration of gut microbial diversity, reinforcement of intestinal barrier integrity via tight junction protein upregulation (e.g., ZO-1 and occludin), and normalization of BA/SCFA metabolism. Among the 10 botanical drugs and 11 formulations reviewed, significant reduction in liver steatosis were shown in rodent models. However, only 4% of these interventions progressed to human trials, and critical methodological inconsistencies were observed, including inconsistent phytochemical standardization and overreliance on homogeneous animal models (68% using male C57BL/6 mice).

**Conclusion:**

While TCM shows promise in modulating microbiota-liver crosstalk, clinical translation is hindered by insufficient phytochemical standardization, unvalidated multi-component synergies, and a paucity of human efficacy data. To bridge this gap, future research must prioritize randomized controlled trials with liver histology endpoints, ConPhyMP-guided quality control protocols, and humanized microbiota models. Rigorous validation of TCM’s microbiota-centric mechanisms—rather than empirical applications—will be essential to advance these interventions into clinically actionable therapies for MASLD.

## 1 Introduction

### 1.1 Current situation of MASLD

Metabolic dysfunction-associated steatotic liver disease (MASLD) was previously referred to as non-alcoholic fatty liver disease (Rinella et al., 2023; De et al., 2024). MASLD encompasses a spectrum from hepatic steatosis to metabolic dysfunction-associated steatohepatitis (Ueno et al., 2013), and may progress to cirrhosis and hepatocellular carcinoma (HCC). Beyond its direct hepatotoxic effects, MASLD demonstrates strong epidemiological associations with type 2 diabetes mellitus (T2DM), cardiovascular diseases (CVD), and chronic kidney disease (CKD) (Targher et al., 2021).

Contemporary research classifies MASLD as a multisystem metabolic disorder with established links to insulin resistance and genetic predisposition. The escalating global pandemic of obesity and its associated metabolic comorbidities are driving MASLD prevalence to unprecedented levels, with projections indicating a surge to 55.4% globally by 2040 (Le et al., 2022). Epidemiological modeling by Estes et al. reveals China’s distinctive MASLD profile, exhibiting the youngest onset age quartile combined with the most pronounced absolute and relative prevalence growth rates. Projections suggest a 29.1% increase in MASLD cases from 2016 baseline levels by 2030, potentially resulting in substantial socioeconomic burdens through direct healthcare costs and productivity losses (Estes et al., 2018).

### 1.2 The relationship between the gut microbiota and MASLD

The human gastrointestinal tract harbors a complex microbial ecosystem that critically regulates host physiological homeostasis through metabolic and immunomodulatory cross-talk. At the core of this symbiotic relationship lies the bidirectional gut-liver axis, where the portal circulation serves as a conduit for continuous molecular exchange: gut-derived microbial metabolites are transported to the liver for processing, while hepatobiliary secretions, particularly bile acids (Misrani et al., 2024), reciprocally regulate intestinal microbial composition and barrier function (Singh et al., 2023).

Recent advancements in MASLD pathogenesis research have progressively unraveled the critical role of gut microbiota dysbiosis within this axis (Rong et al., 2022). Longitudinal cohort studies demonstrate stage-specific microbial alterations across the MASLD spectrum, characterized by distinct taxonomic and functional shifts that mirror disease progression. During early-stage hepatic steatosis, metagenomic analyses reveal enrichment of butyrate-producing taxa such as *Eubacterium rectale* and *Bacteroides vulgatus*, which may initially compensate for metabolic stress by enhancing short-chain fatty acid (SCFA) production and maintaining intestinal barrier integrity (Loomba et al., 2017). However, as steatosis progresses to MASH, a marked reduction in microbial diversity emerges, with proliferation of endotoxin-producing Proteobacteria (e.g., *Escherichia coli*, *Escherichia-shigella*), exacerbating intestinal permeability and systemic inflammation (Frost et al., 2021; Cornejo-Pareja et al., 2024). As disease progresses to cirrhosis and its associated complications, the changes in microbiome become even more stark. With advancing disease, there is evidence of higher potential pathobionts (*Enterobacteriaceae*, *Enterococcaceae*), and lower relative abundance of commensal taxa, creating a microenvironment conducive to hepatocyte injury and fibrogenesis (Lang et al., 2020; Saeed et al., 2025). In addition to the microbial structure, multi-omics studies have identified stage-specific microbial metabolites, including BAs also change with liver disease progression (Puri et al., 2018; McGlinchey et al., 2022). This temporal dysbiosis-MASLD trajectory underscores the gut-liver axis as both a driver and biomarker of disease progression. The shift from compensatory metabolic adaptations to persistent microbiota-driven inflammation highlights therapeutic opportunities for stage-specific microbiome modulation, potentially interrupting the pathogenic continuum before irreversible fibrotic remodeling occurs (Fang et al., 2022b).

### 1.3 Microbiome-targeted interventions in MASLD management

Current therapeutic frameworks for nonalcoholic fatty liver disease (MASLD) remain suboptimal, necessitating novel approaches to halt disease progression. While the AASLD guidelines emphasize lifestyle modification as foundational therapy, pharmacologic options—including vitamin E, pioglitazone, and ursodeoxycholic acid (UDCA)—demonstrate limited histological benefits and are not universally recommended for non-biopsy-confirmed cases (Chalasani et al., 2018). This therapeutic impasse has catalyzed exploration of microbiota-centric interventions, leveraging emerging insights into gut-liver axis pathophysiology (Saeed et al., 2025).

Accumulating clinical evidence positions microbiome modulation as a promising adjunctive strategy. A meta-analysis of 13 randomized controlled trials revealed that probiotic/prebiotic/synbiotic supplementation significantly improves key MASLD parameters: reducing hepatic fat fraction, attenuating systemic inflammation, and restoring the gut microbiota (Carpi et al., 2022). Fecal microbiota transplantation (FMT), though investigational, demonstrates therapeutic potential through ecological reconstitution. Several studies have shown that FMT can reduce fat deposition by improving intestinal permeability and gut microbiota disorders in MASLD patients (Craven et al., 2020; Xue et al., 2022). The therapeutic potential of bacteriophage therapy is being explored and has been shown to specifically edit the gut microbiota (Saeed et al., 2025).

It was suggested that colonization of the gut by high-alcohol-producing *Klebsiella pneumoniae* (HiAlc Kpn) may contribute to MASLD through the production of ethanol. Further studies found that healthy mice transplanted with the gut microbiota of MASLD mice harboring HiAlc Kpn developed significant steatosis 4 weeks after gavage, whereas hepatic steatosis was significantly alleviated after pretreatment with bacteriophage phiW14/TH1-302 (Yuan et al., 2019). Subsequent investigations showed that bacteriophage specialized in eradicating HiAlc Kpn not only alleviated hepatic steatosis, but also reprogrammed the gut microbiota without significant side effects (Gan et al., 2023). Microbiome therapy provides a new avenue for the treatment of MASLD, and although it shows promise, more in-depth research is needed to complete the transition from experimental to clinical studies for the clinical treatment of MASLD.

## 2 Literature search strategy

A systematic literature search was conducted across PubMed, Web of Science, ScienceDirect, Google Scholar, and the China National Knowledge Infrastructure (CNKI) database to identify relevant studies published up to May 2025. Search terms included controlled vocabulary (MeSH terms) and free-text keywords such as “gut microbiota,” “Traditional Chinese Medicine,” “metabolic dysfunction-associated steatotic liver disease (MASLD),” “non-alcoholic fatty liver disease (MASLD),” “metabolic dysfunction-associated steatohepatitis (Ueno et al.),” “intestinal barrier,” “bile acid metabolism,” “TLR4 signaling,” “FXR-FGF15 axis,” and “botanical metabolites.” Boolean operators (AND/OR) were applied to combine terms, and database-specific filters (e.g., publication type, language, species) were used to refine results. Inclusion criteria prioritized original research articles and reviews addressing gut microbiota modulation by botanical metabolites and polyherbal formulations in MASLD, with emphasis on mechanistic studies using validated *in vivo* or *in vitro* systems. Studies lacking experimental validation (e.g., purely descriptive analyses), non-peer-reviewed publications, or those with insufficient methodological detail (e.g., undefined herbal formulations, unclear dosing regimens) were excluded. Duplicate records were removed, and remaining articles underwent title/abstract screening followed by full-text evaluation for eligibility. Reference lists of key papers were manually searched to identify additional relevant studies.

## 3 Gut microbiota in MASLD pathogenesis

**FIGURE 1:**
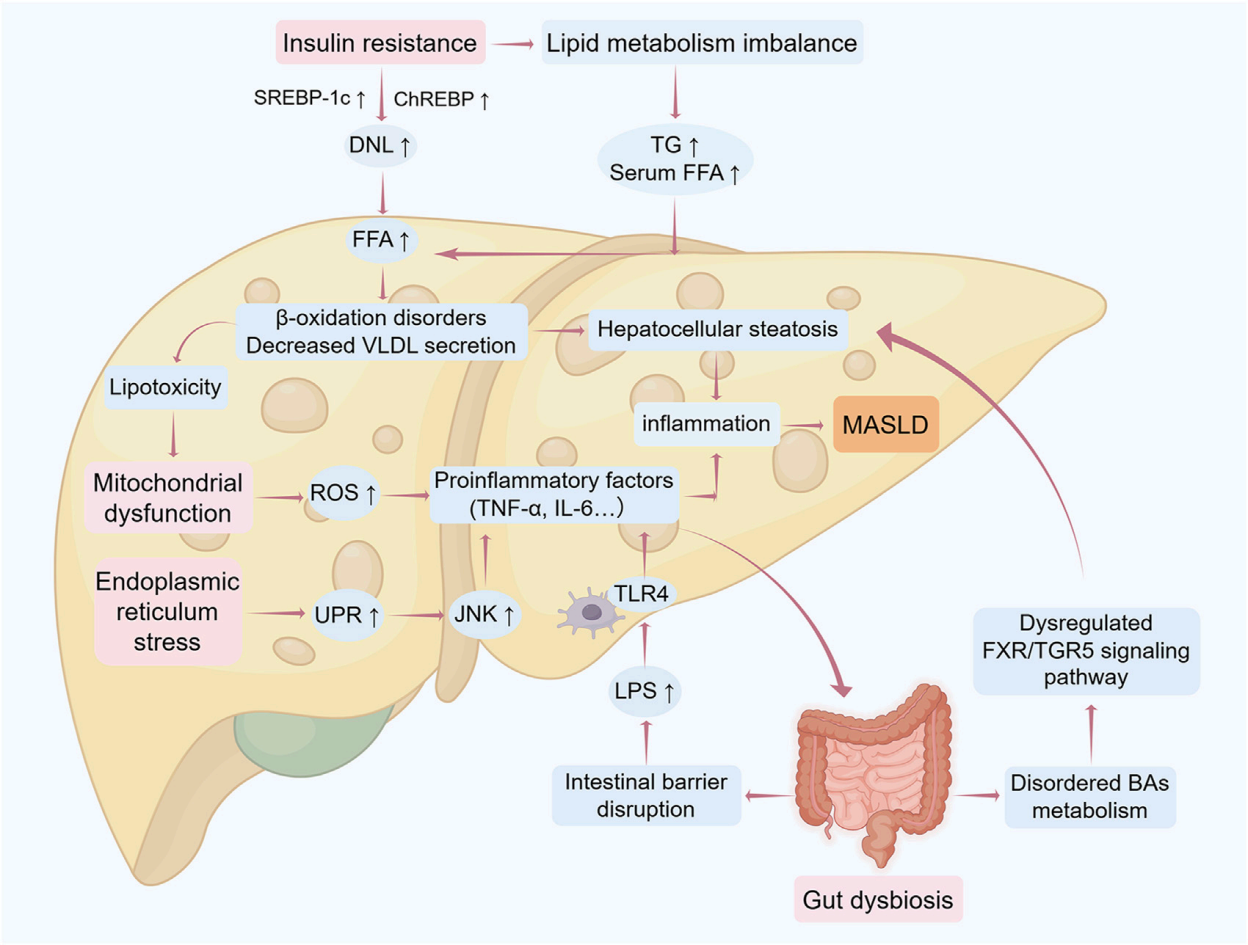
Mechanistic framework of the “multiple-hit” hypothesis in MASLD progression The pathogenesis of MASLD involves sequential pathological hits driving progression from hepatic steatosis to end-stage liver injury. Initial metabolic insults: Insulin resistance triggers peripheral lipolysis, increasing free fatty acid (Lena et al.) influx into the liver. Concurrently, enhanced *de novo* lipogenesis (DNL), impaired fatty acid β-oxidation, and reduced very low-density lipoprotein (VLDL) secretion collectively promote triglyceride (TG) accumulation, initiating simple steatosis. Oxidative and ER stress: Excessive FFA overload induces mitochondrial dysfunction and reactive oxygen species (ROS) overproduction, exacerbating oxidative stress and unfolded protein response (UPR)-mediated endoplasmic reticulum stress. These processes activate c-Jun N-terminal kinase (JNK) and nuclear factor-κB (NF-κB) pathways, releasing pro-inflammatory cytokines (e.g., TNF-α, IL-6), thereby transitioning steatosis to steatohepatitis. Gut-liver axis dysregulation: Gut microbiota dysbiosis disrupts intestinal barrier integrity, enabling lipopolysaccharide (LPS) translocation into the liver. Hepatic TLR4 activation amplifies inflammatory cascades and fibrotic signaling. Bile acid (BA) dysmetabolism: Aberrant FXR/TGR5 signaling due to BA imbalance further disrupts hepatic metabolic homeostasis, aggravating hepatocyte injury and disease progression. *Abbreviations*: DNL, *de novo* lipogenesis; ER, endoplasmic reticulum; FFA, free fatty acids; FXR, Farnesoid X receptor; IL-6, interleukin 6; JNK, c-Jun N-terminal kinase; LPS, lipopolysaccharide; MASLD, metabolic dysfunction-associated steatotic liver disease; ROS, reactive oxygen species; TG, triglycerides; TLR4, Toll-like receptor 4; TNF-α, tumor necrosis factor alpha; UPR, unfolded protein response; VLDL, very low-density lipoproteins. The figure was created using Figdraw.

The conceptual framework of MASLD pathogenesis has evolved from the “two-hit hypothesis” to a “multiple-hit paradigm” through progressive scientific inquiry. Initially, the first hit postulated hepatic lipid metabolism dysregulation, emphasizing insulin resistance and adipocytokine imbalances involving lipocalin and leptin. Subsequently, the second hit expanded this model to incorporate steatosis progression mechanisms, including endoplasmic reticulum stress, oxidative stress, and fibrotic transformation (Teng et al., 2022). As shown in Figure 1, contemporary research transcends these models through the multiple-hit hypothesis, which systematically integrates: 1) metabolic dysregulation (insulin resistance, lipotoxicity), 2) cellular stress responses (mitochondrial dysfunction, inflammation), and 3) microenvironmental modulators (hormonal factors, gut microbiota, epigenetic regulators) (Buzzetti et al., 2016).

Among these factors, there is growing evidence that the gut microbiota and its derivatives, the intestinal mucosal barrier, etc., play a key role in the pathogenesis of MASLD. The gut microbiota has been shown to be involved in MASLD progression through metabolites such as LPS, SCFAs and BAs. The intestinal mucosal barriers are the body’s vital defense against harmful substances and infectious agents, and any damage to these barriers may lead to intestinal dysfunction, triggering intestinal infections, flora disorders, promoting liver inflammation, and ultimately leading to the development of MASLD.

### 3.1 Intestinal mucinal barrier dysfunction in MASLD pathogenesis

The intestinal mucosal barrier comprises four functionally integrated components: mechanical, chemical, immune, and biological barriers. The primary defense layer consists of intestinal epithelial cells (IECs) interconnected through tight junctions (TJs), overlaid by a mucus bilayer. This architecture selectively restricts bacterial translocation and endotoxin infiltration into systemic circulation (Albillos et al., 2020). Epithelial cellular diversity–encompassing goblet cells, Paneth cells, tuft cells, and microfold (M) cells–extends beyond structural roles. These specialized cells actively secrete mucins, antimicrobial peptides (Sampson et al., 2016), and trophic factors that collectively maintain mucosal integrity and coordinate barrier functions (Sardinha-Silva et al., 2022). Goblet cell-derived mucin (MUC2) forms a stratified mucus structure with distinct dense and loose layers, establishing critical spatial segregation between luminal microbiota and epithelial surfaces (Faderl et al., 2015). This glycoprotein matrix entraps digestive enzymes, AMPs, and secretory IgA (sIgA), creating a biochemical defense system through microbial degradation and cell wall disruption (Kayama et al., 2020; An et al., 2022). Even if microorganisms in the intestinal lumen break through the intestinal epithelium into the lamina propria, there they will face the defense of the immune barrier. The immune barrier includes both humoral and cellular immunity, and humoral factors such as AMP and sIgA, which limit the colonization and growth of pathogenic microorganisms, protect the intestinal mucosa from damage (Hrncir et al., 2021). Immune cells such as macrophages, dendritic cells, and innate lymphocytes are able to fight potential pathogens and protect mucosal integrity (Chopyk and Grakoui, 2020). The intestinal biobarrier is a microecosystem composed of intestinal resident bacteria and host microspace structures that provide colonization resistance to potential pathogens (Wells et al., 2017). In addition, gut commensal microorganisms indirectly enhance barrier protection by stimulating pathogen recognition receptors on epithelial cells as well as promoting adaptive immunity (Chopyk and Grakoui, 2020). It is evident that intestinal barriers are interconnected and influential.

Intestinal barrier dysfunction initiates a pathogenic cascade through increased mucosal permeability, enabling bacterial translocation via the portal circulation to hepatic tissue. This process activates hepatic immune responses that exacerbate parenchymal injury. Clinical evidence demonstrates that MASLD patients exhibit significant intestinal mucosal damage with elevated permeability compared to healthy controls, showing strong positive correlations with hepatic inflammatory activity (p < 0.05) and fibrosis (p < 0.001) (Giorgio et al., 2014). The pathogenesis involves multifactorial interactions, with HFD and gut dysbiosis representing predominant etiological factors. Chronic HFD exposure induces disruption of TJ protein complexes while exacerbating microbiota-mediated barrier dysfunction (Nian et al., 2024). Proteomic analysis of IECs revealed HFD-induced TJ dysregulation, demonstrating that dietary lipid overload drives MASLD progression through TJ-mediated elevation of intestinal permeability (Muto et al., 2023). Perturbations in the intestinal luminal environment significantly contribute to enhanced paracellular permeability. BAs, key components of the enterohepatic circulation, are essential regulators of intestinal homeostasis. Excessive BA concentrations exhibit direct cytotoxicity to intestinal epithelia, compromising mucosal integrity and promoting bacterial translocation - a critical mechanism facilitating MASLD progression to MASH (Gupta et al., 2020). Thus, any factor that alters the above gut barrier components will result in a compromised intestinal mucosal barrier, which will allow gut microbial metabolites and toxins to translocate to the liver through the portal vein, activate the immune response, and increase the risk of MASLD development.

### 3.2 Effect of gut microbiota-derived metabolites on MASLD

#### 3.2.1 SCFAs

The gut microbiota metabolizes undigested dietary components into SCFAs, primarily acetate (60%–70%), propionate (15%–20%), and butyrate (10%–15%), which collectively regulate intestinal barrier integrity, metabolic homeostasis, and immune function (Morrison and Preston, 2016). Acetate serves as both an energy substrate and signaling molecule, with experimental studies demonstrating its capacity to activate hepatic free fatty acid receptor 2 (FFAR2), thereby improving insulin sensitivity and attenuating lipid accumulation in MASLD models (Aoki et al., 2021). Butyrate exerts multifaceted protective effects through enhancing TJ protein expression, and inhibiting macrophage activation and inflammatory factor production, thereby reducing endotoxin translocation and achieving attenuation of secondary liver injury (Liu et al., 2014). SCFAs further reinforce intestinal barrier function by stimulating interleukin-18 (IL-18) secretion through NLRP3 inflammasome activation, while their portal circulation-mediated hepatic delivery directly suppresses lipogenic pathways via sterol regulatory element-binding protein 1c (SREBP1c) downregulation (Macia et al., 2015; Dai et al., 2020).

Dysbiosis-induced SCFA depletion exacerbates MASLD progression through impaired glucagon-like peptide-1 (GLP-1) secretion, elevated intestinal permeability, and aggravated endotoxemia. Preclinical evidence indicates that high-fat diet-fed mice exhibit marked reductions in SCFA-producing bacteria and corresponding metabolite levels, which correlate with accelerated hepatic steatosis and fibrosis (Huang et al., 2020). Therapeutic restoration of SCFAs through dietary modulation or direct supplementation demonstrates significant metabolic benefits, including reduction in hepatic triglyceride content and decrease in fibrotic markers, highlighting their potential as therapeutic targets in MASLD management (Baumann et al., 2020; Zhao et al., 2021).

#### 3.2.2 BAs

BAs undergo hepatic biosynthesis from cholesterol followed by conjugation with taurine/glycine prior to biliary secretion. Approximately 95% of secreted BAs are reabsorbed through ileal enterocytes, completing the enterohepatic circulation - a process critically modulated by gut microbial biotransformation. Intestinal microbiota mediate BA deconjugation and 7α-dehydroxylation, converting primary BAs (cholic acid [CA], chenodeoxycholic acid [CDCA]) into secondary forms (deoxycholic acid [DCA], lithocholic acid [LCA]) with distinct physicochemical properties (Tilg et al., 2022). Clinical metabolomic analyses reveal MASLD patients exhibit 4-fold elevated serum DCA compared to healthy controls, establishing BA dysmetabolism as a hallmark of disease progression (Jiao et al., 2018).

The signaling potency of BAs is governed by receptor specificity: FXR demonstrates nanomolar affinity for CDCA versus millimolar responses to DCA, while G-protein-coupled BA receptor, Gpbar1 (TGR5) is preferentially activated by LCA (Inagaki et al., 2006; Safari and Gerard, 2019; Merlen et al., 2020). This ligand-receptor selectivity implies that dysbiosis-induced BA compositional shifts can dysregulate hepatic metabolism through altered FXR/TGR5 signaling cascades. Mechanistically, FXR activation preserves intestinal barrier function by upregulating tight junction proteins and suppressing bacterial translocation, whereas TGR5 signaling modulates macrophage polarization towards anti-inflammatory phenotypes (Yamada et al., 2018; Wu et al., 2021).

#### 3.2.3 LPS

Lipopolysaccharide (LPS), a glycolipid constituent of Gram-negative bacterial membranes, functions as a potent pathogen-associated molecular pattern that drives chronic low-grade inflammation in MASLD pathogenesis (Ji et al., 2019). Hepatic recognition of circulating LPS occurs through a multi-receptor complex comprising Toll-like receptor 4 (TLR4), myeloid differentiation protein-2 (MD-2), and co-receptor CD14, with lipopolysaccharide-binding protein (LBP) facilitating endotoxin transfer to this signaling complex. Activation of this pathway in Kupffer cells triggers NF-κB-mediated proinflammatory cytokine production, establishing a microenvironment conducive to MASLD progression (Ferro et al., 2020; Wang L. et al., 2024). Besides, LPS activates the NLRP3 inflammasome via TLR4 signaling in adipocytes, triggering IL-1β/IL-18 release that drives adipose tissue inflammation and fibrosis. Experimental NLRP3 inhibition suppresses LPS-induced proinflammatory cytokine production and downregulates collagen/extracellular matrix (ECM) remodeling genes, demonstrating its dual therapeutic potential for mitigating metabolic inflammation and fibrotic progression in obesity-related disorders (Liu et al., 2016; Unamuno et al., 2021).

Beyond its pro-inflammatory effects, LPS demonstrates dual regulatory roles in liver pathophysiology. LBP deficiency exacerbates hepatic steatosis despite attenuating inflammation - evidenced by excessive fat deposition in LBP−/− mice compared to wild-type controls (Zhu et al., 2024). This dichotomy underscores the complex interplay between endotoxin signaling and metabolic regulation. The intestinal barrier emerges as both source and target of LPS-mediated injury. Physiological concentrations of LPS compromise TJ integrity through TLR4-dependent process, creating a vicious cycle of endotoxemia and barrier dysfunction (Guo et al., 2013).

#### 3.2.4 Endogenous ethanol

The gut microbiota serves as the primary source of endogenous ethanol. Zhu et al. (2013) conducted a comparative analysis of gut microbial composition and peripheral blood ethanol levels among children with MASH, obesity, and healthy controls. Their findings revealed significant alterations in gut microbiota profiles in both obese and MASH groups compared to healthy individuals, with MASH patients exhibiting elevated abundances of ethanol-producing bacterial taxa and markedly higher circulating ethanol concentrations. These observations imply that microbiota-derived endogenous ethanol may function as a hepatotoxic agent contributing to MASLD initiation and progression.

Emerging evidence has elucidated mechanistic links between endogenous ethanol and MASLD pathogenesis. First, ethanol upregulates cytochrome P450 2E1 (CYP2E1) enzymatic activity, amplifying reactive oxygen species (ROS) generation. This process induces hepatic oxidative stress (OS) and inflammatory cascades, thereby exacerbating hepatocellular injury and accelerating MASLD progression (Aljomah et al., 2015; Abdelmegeed et al., 2017). Second, ethanol directly impairs mitochondrial function in hepatocytes, a hallmark of advanced MASLD (Chen X. et al., 2020). Third, ethanol exacerbates liver damage indirectly through its metabolite acetaldehyde, which induces oxidative stress and disrupts intercellular TJs (Liu et al., 2022). As demonstrated by Dunagan et al. (2012), acetaldehyde-mediated TJ dysfunction increases intestinal paracellular permeability, facilitating translocation of microbial components or metabolites into the portal circulation. This process triggers systemic inflammation and further aggravates hepatic injury, establishing a vicious cycle in MASLD progression.

#### 3.2.5 Choline

Choline, an essential nutrient predominantly sourced from dietary components including red meat, eggs, and nuts, plays a critical role in hepatic lipid metabolism and regulates BAs enterohepatic circulation (Xiang et al., 2022). Emerging evidence implicates choline deficiency as a contributor to MASLD progression. As a key substrate for very-low-density lipoprotein (VLDL) biosynthesis, insufficient choline availability reduces hepatic VLDL secretion, leading to triglyceride (TG) accumulation and subsequent hepatocyte injury (Jacob et al., 2021). This mechanistic insight underpins the widespread use of methionine-choline-deficient (MCD) diets to induce MASH in preclinical models, where resultant phenotypes are closely associated with intestinal inflammatory responses (Matthews et al., 2021).

Gut microbiota-mediated metabolism further links choline to MASLD pathogenesis. Microbial conversion of choline to trimethylamine (TMA) is followed by hepatic oxidation via flavin-containing monooxygenase 3 (FMO3), generating trimethylamine N-oxide (TMAO). Clinical studies demonstrate elevated TMAO levels correlating with MASLD severity and MASH risk in humans (Leon-Mimila et al., 2021). Experimental models reveal that TMAO administration exacerbates hepatic steatosis by enhancing *de novo* lipogenesis and impairing BA-activated FXR signaling (Tan et al., 2019). Furthermore, TMAO aggravates metabolic dysregulation through multiple pathways: disrupting intestinal barrier integrity, compromising hepatic endothelial function, and modulating macrophage polarization toward proinflammatory phenotypes (Nian et al., 2024). While these findings highlight TMAO’s pathogenic role, its potential as a therapeutic target for MASLD warrants rigorous investigation.

#### 3.2.6 Amino acids

Hepatic disturbances in amino acid and lipid metabolism promote fatty acid deposition, triggering OS and hepatocellular injury that accelerates MASLD progression (Deng et al., 2024). Comparative analyses of serum amino acid profiles among healthy controls, MASLD patients, and hepatic fibrosis cases revealed elevated circulating levels of branched-chain amino acids (BCAAs), glutamic acid (Ekstrand et al., 2017), and alanine (Ala) in fibrosis patients, with MASLD subjects showing particularly pronounced Ala elevation. These metabolic alterations demonstrate strong correlations with insulin resistance and hepatic metabolic dysfunction (Hasegawa et al., 2020).

Mechanistic studies elucidate BCAA-mediated hepatotoxicity through dual tissue-specific pathways. In adipocytes, BCAAs activate AMPKα2 to stimulate lipolysis, thereby increasing plasma free fatty acid (Lena et al., 2005) release. Concurrently, hepatic BCAAs activate mammalian target of rapamycin (mTOR) signaling, which inhibits FFA-to-TG conversion, exacerbates FFA-induced lipotoxicity, suppresses hepatocyte autophagy, and promotes apoptosis, collectively driving liver injury (Zhang et al., 2016).

Aromatic amino acids (AAAs) - tryptophan (Trp), phenylalanine (Phe), and tyrosine (Tyr) - exert bidirectional regulatory effects on MASLD pathogenesis. Hepatic Trp metabolism via tryptophan 2,3-dioxygenase (TDO2) and indoleamine 2,3-dioxygenase (IDO) generates kynurenine (Kyn), which activates aryl hydrocarbon receptor (AHR) signaling to promote obesity-associated hepatic steatosis (Rojas et al., 2021). Conversely, gut microbiota metabolize Trp into protective indole derivatives including indole-3-acetic acid (IAA), indole-3-propionic acid (IPA), and tryptamine (Dai et al., 2020). Notably, IPA demonstrates therapeutic potential by ameliorating gut dysbiosis, enhancing intestinal barrier integrity to prevent endotoxin translocation, and suppressing NF-κB-mediated proinflammatory cytokine release (Zhao et al., 2019).

These findings collectively highlight that dysregulated amino acid metabolism - through both hepatotoxic and protective pathways - constitutes a key mechanistic axis in MASLD development. Targeted modulation of these metabolic networks may yield novel therapeutic strategies for MASLD management.

**FIGURE 2:**
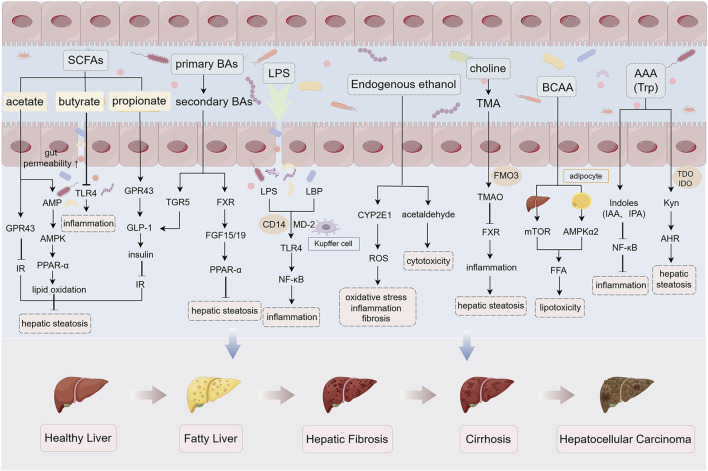
Gut microbiota-derived metabolites in MASLD pathogenesis under dysbiosis. Under gut microbiota dysbiosis, multiple microbial metabolites—including SCFAs, BAs, LPS, endogenous ethanol, choline, and amino acids—are dysregulated, collectively driving MASLD progression. SCFAs (acetate, propionate, butyrate): Attenuate hepatic steatosis by activating AMPK and GPR signaling while suppressing TLR4-mediated inflammation. Dysbiosis reduces SCFA production, exacerbating intestinal barrier dysfunction and hepatic lipid deposition. Secondary BAs: Inhibit hepatocyte steatosis via TGR5/FXR signaling activation, whereas dysmetabolism of primary Bas promotes barrier disruption. LPS: Triggers hepatocyte inflammation and fibrosis through TLR4/NF-κB pathway activation. Endogenous ethanol: Upregulates CYP2E1 to induce oxidative stress and acetaldehyde-mediated hepatotoxicity. Choline-TMA/TMAO axis: Microbial conversion of choline to trimethylamine (TMA) and subsequent hepatic oxidation to TMAO suppresses FXR signaling, aggravating steatosis. Amino acids: BCAAs promote lipotoxicity via dual regulation of adipocyte lipolysis and hepatic mTOR signaling; AHR-activating Kyn exacerbates steatosis, while gut microbiota-derived indole derivatives enhance barrier integrity and suppress inflammation. *Abbreviations*: AMPK, AMP-activated protein kinase; GPR, G-protein-coupled receptor; TLR4, Toll-like receptor 4; TGR5, G-protein-coupled bile acid receptor 1; FXR, Farnesoid X receptor; CYP2E1, cytochrome P450 2E1; TMA, trimethylamine; TMAO, trimethylamine N-oxide; mTOR, mammalian target of rapamycin. The figure was created using Figdraw.

In summary, in the condition of gut microbiota dysbiosis, various bacterial metabolites such as SCFAs, Bas, LPS, endogenous ethanol, choline, and amino acids are altered and involved in the pathogenesis of MASLD (Figure 2).

## 4 Targeting gut-liver axis: the role of TCM in MASLD through microbiota modulation

**FIGURE 3:**
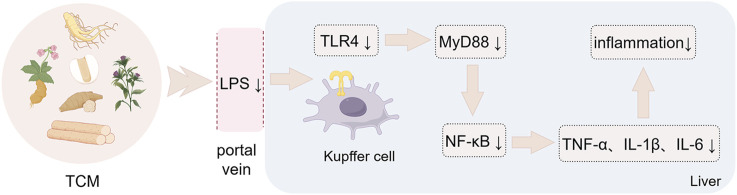
Botanical drugs-mediated immunomodulation through LPS/TLR4 signaling. Botanical drugs and metabolites against MASLD by regulating intestinal immunity through LPS/TLR4 signaling pathway. The figure was created using Figdraw.

**FIGURE 4:**
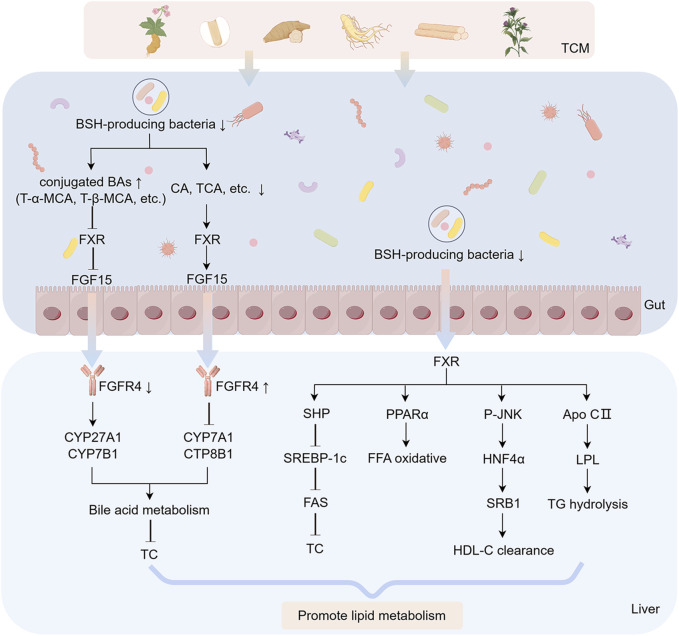
FXR-FGF15 axis modulation through botanical drug-mediated BA regulation. Botanical drugs and metabolites against MASLD by regulating BAs metabolism through FXR-FGF15 signaling axis. The figure was created using Figdraw.

The pathogenesis of MASLD is driven by a complex interplay of metabolic dysregulation, gut-liver axis disruption, and microbiota-derived inflammatory signaling. As outlined in preceding sections, hepatic lipid accumulation and inflammatory cascades are exacerbated by gut microbiota dysbiosis, characterized by diminished microbial diversity, impaired intestinal barrier integrity, and altered metabolite profiles (e.g., reduced SCFAs, elevated LPS, and dysregulated BA metabolism). These perturbations activate key pathways, including TLR4/NF-κB-mediated inflammation, FXR-FGF15 axis dysfunction, and oxidative stress, collectively propelling disease progression from steatosis to fibrosis. Despite advancements in understanding these mechanisms, current therapeutic strategies remain limited in efficacy, underscoring the need for innovative interventions targeting the gut-liver axis. TCM has emerged as a promising candidate. Preclinical evidence highlights TCM’s capacity to restore microbial homeostasis, enhance intestinal barrier function, and modulate critical metabolites and signaling pathways. The following sections elucidate how botanical drugs and polyherbal formulations mitigate MASLD through microbiota-centric mechanisms, bridging ancient therapeutic wisdom with modern molecular insights (Tables 1, 2; Figures 3, 4).

**TABLE 1:** Metabolites of botanical drugs for the treatment of MASLD.

Sources information	Plant metabolites	Study Type	Experiment Object (n)	Dosage and Intervention mode	Modeling methods	Positive control group
Cassia obtusifolia L. [Fabaceae; Cassiae Semen]	Aurantio-obtusin	*In vivo*	Male *ApoE* ^*−/−*^ C57BL/6 mice; Wild- type mice (30, 6 mice/group)	Low/High-dose group: 10/20 mg/kg, gavage 16 weeks	High-fat diet	ATO: atorvastatin 10 mg/kg, gavage 16 weeks
*Cassia obtusifolia* L. [Fabaceae; Cassiae Semen]	Chrysophanol, Aurantio-obtusin	*In vivo*	Male C57BL/6 mice (60, 10 mice/group)	10 g/kg, gavage 3 weeks	High-fat diet	Total aglycone (TA): 10 g/kg, gavage 3 weeks; Rubrofusarin-6-β-gentiobioside (RG): 20 mg/kg, gavage 3 weeks; Aurantio-obtusin (AO): 20 mg/kg, gavage 3 weeks
*Coptis chinensis* Franch. [Ranunculaceae; Coptidis Rhizoma]	Berberine	*In vivo*	Male C57BL/6J mice; Male FXR^−/−^ C57BL/6J mice (24, 8 mice/group)	100 mg/kg, gavage 4 weeks	High-fat diet with interval dextran sulfate sodium (0.5% in drinking water)	_
*Pueraria lobata* (Willd.) Ohwi [Fabaceae; Puerariae Lobatae Radix]	Puerarin	*In vivo*	Male C57BL/6 mice (18, 6 mice/group)	0.2 g/kg, gavage 4 weeks	MCD diet	_
*Pueraria lobata* (Willd.) Ohwi [Fabaceae; Puerariae Lobatae Radix]	Puerarin, Daidzin, Daidzein	*In vivo*	Male C57BL/6J mice (24, 6 mice/group)	400 mg/kg, oral administration 8 weeks	High-fat diet	Silymarin: 100 mg/kg, every other day, gavage 8 weeks
*Ophiopogon japonicus* (Thunb.) Ker Gawl. [Asparagaceae; Ophiopogonis Radix]	Water-soluble inulin-type β-D-fructan	*In vivo*	Male C57BL/6J mice (40, 8 mice/group)	Low/Middle/High-dose MDG-1 group: 2‰/4‰/8‰ MDG-1 mixed into the high-fat diet, oral administration 8 weeks	High-fat diet	_
*Dendrobium officinale* Kimura et Migo [Orchidaceae; Dendrobii officinalis Caulis]	Polysaccharides, Phenanthrenes, Bibenzyls	*In vivo*	Male Sprague-Dawley rats (48, 8 rats/group)	Low/Middle/High dose DO group: 250/500/1,000 mg/kg, gavage 10 weeks	High-fat diet	Atorvastatin calcium: 20 mg/kg, gavage 10 weeks
*Astragalus membranaceus* Fisch. ex Bunge [Fabaceae; Astragali Radix]	Astragalus polysaccharide	*In vivo*	Male Sprague–Dawley rats (50, 10 rats/group)	200 mg/kg, gavage 4 weeks	High-fat diet	Berberine: 300 mg/kg, gavage 4 weeks
*Astragalus membranaceus* Fisch. ex Bunge [Fabaceae; Astragali Radix]	Astragaloside Ⅳ	*In vivo*	Male C57BL/6N mice (48, 8 mice/group)	Low/Middle/High dose group: 12.5/25/50 mg/kg, gavage 12 weeks	High-fat diet	Fenofibrate
*Schisandra chinensis* Fructus [Schisandraceae; Schisandrae Chinensis Fructus]	Schisantherin A	*In vivo*	Male C57BL/6J mice	80 mg/kg, gavage 6 weeks	High-fat diet	TLR4 inhibitor: 2 mg/kg, *i.p*. injection
*Apis mellifera* Linnaeus [Apidae; Vespae Nidus]	Caffeic acid phenethyl ester	*In vivo*	Wild-type mice, Fxr^fl/fl^ mice and intestine-specific Fxr-null (Fxr^ΔIE^) mice	75 mg/kg, gavage 8 weeks	High-fat diet	_
Vitis, Ampelopsis, Polygonum, Arachis, Veratrum, etc.	Resveratrol	*In vivo*	Male Sprague–Dawley rats	Low/High-Resveratrol group: 50/100 mg/kg, gavage 6 weeks, High-Resveratrol group gavage 12 weeks	High-fat diet	_
*Belamcanda chinensis* (L.) Redouté [Iridaceae; Belamcandae Rhizoma]	Tectorigenin	*In vivo*	Male C57BL/6N mice (24, 6 mice/group)	Low/High Tg group: 25/50 mg/kg, gavage 6 weeks	High-fat diet	_

Effects on gut microbiota	Effects on intestinal mucosal barrier	Gut microbiota-derived metabolites	Metabolic phenotypes	Mechanisms	References
↑ g_*Desulfovibrio* and g_*Lach noclostridium;* ↑ g_*unclassified*_*f*_*Muribaculaceae,* g_*Lachnospiraceae*_*NK4A136*_*group*; ↓ g_*Allobaculum*; ↓ *Firmicutes*/*Bacteroidetes* ratio	↑ Tight junctions between epithelial cells; ↑ Goblet cell numbers; ↑ Viscous substance content; ↑ ZO- 1, occludin, and claudin-1	_	↓ Weight; ↓ TC, TG, and LDL-C; ↓ Visceral adiposity and subcutaneous adiposity indices	↑ PPARα and CPT1A; ↓ SREBP1, FASN and SCD1	Li et al. (2025)
↑ *Dehalobacterium*, *Oscillospira*, *Coprococcus*, and *Ruminococcus;* ↓ *Proteobacteria*, *Enterobacteriaceae*, *Erwinia*, *Klebsiella*, *Morganella*, and *Trabulsiella*	↑ ZO-1 and occludin	↓ LPS	↓ Liver weight index; ↓ TC, TG, FFA, and LDL-C	Regulate the gut microbiota	Luo et al. (2021)
↑ *Bacteroidetes*; ↑ *Clostridiales*, *Lactobacillaceae*, and *Bacteroidale*	_	↓ Serum and liver BA; ↑ Fecal TBA; ↑ Fecal CDCA, UDCA, DCA, and MCA; ↓ Fecal TβMCA	↓ Body weight and liver weight; ↓ liver TG; ↓ Serum TG, TC, LDL-C; ↓ Serum ALT, AST	Activation of intestinal FXR-FGF15 signaling	Shu et al. (2021)
↓ *Epsilonbacteraeota*; ↑ *Roseburia*; ↓ *Helicobacter*	_	_	↓ Body weight; ↓ Serum ALT and AST; ↓ Liver TG	_	Gong et al. (2021)
↓ *Firmicutes*/*Bacteroidetes* ratio; ↓ *Desulfobacterota*; ↑ *Lactobacillus*, *Bifidobacterium*, *Turicibacte*r and *Actinobacteriota*	Enhancing the stability of intestinal mucosal barrier by increasing the production of SCFA	↑ SCFA (acetic acid, butyric acid, valeric acid, isovaleric acid and hexanoic acid)	↓ Serum ALT, AST, TC, TG and LDL-C; ↓ Lipid accumulation	Promotion of SCFA production	Yang et al. (2022)
↓ *Firmicutes*/*Bacteroidetes* ratio; ↓ *Enterorhabdus*, *Turicibacter*, *Clostridium-sensu-stricto-1*, *Tyzzerella* and *Oscillibacter*; ↑ *Ruminiclostridium* and *Rikenella*; ↑ SCFA-producing bacteria (*Butyricimonas* and *Roseburia*)	_	↑ SCFA (acetic acid and valeric acid)	↓ Body weight; ↓ TC, TG	↓ SREBP-1c, FAS and ACC-1; ↑ AMPK, GPR41 and GPR43; Modulate AMPK-SREBP1 signaling pathway	Wang et al. (2019b)
↓ *Proteobacteria*, *Romboutsia*, *Turicibacter*, *Lachnoclostridium*, *Blautia*, *Ruminococcus*_*torques*_*group*, *Sutterella*, and *Escherichia*-*Shigella*; ↑ *Prevotella* and *Alistipes*; ↑ *Lactobacillus*_*acidophilus*	↑ ZO- 1, claudin-1, and occludin	↓ LPS	↓ Body weight, liver weight, and liver index; ↓ TC, TG, LDL-C, ALT, AST, GGT; ↑ HDL-C	↓ TLR4; ↓ IL-6, IL-1β, and TNF-α	Tian et al. (2023)
↓ *Firmicutes*/*Bacteroidetes* ratio; ↑ *Proteobacteria* and *Episilonbacteria*;	↑ ZO- 1 and occludin	_	↓ Body weight, liver index and eWAT weight; ↓ Serum ALT and AST; ↓ Insulin resistance	↑ AMPK and PPAR-α; ↓ SREBP-1; ↓ TLR4, NLRP3 and phosphorylated-NF-κB	Zhong et al. (2022)
↓ *Bacteroides*, *Lactobacillus*, *Streptococcus*, *Enterococcus*, and *Lactococcus*	_	↑ GCDCA, TUDCA, TDCA, and TβMCA; ↓ LCA, DCA, UDCA, CA, CDCA	↓ Body weight, liver weight and eWAT weight; ↓ Liver TG and TC; ↓ ALT and AST	Inhibition of intestinal FXR and its downstream signaling; activation of liver FXR-SHP signaling	Zhai et al. (2022)
↓ *Firmicutes*/*Bacteroidetes* ratio	↑ Occludin	↓ LPS	↓ Liver size, liver weight, and the ratio of liver weight to body weight; ↓ TG, TC, LDL-C, and FFA; ↑ HDL-C	↓ IL-6, IL-1β, TNF-α, and NOS2; Inhibition of LPS/TLR4 signaling	Yu et al. (2022)
↑ *Bacteroides*, *Enterococcus*, *Bilophila* and *Helicobacter*; ↓ *Intestinimonas*, *Lachnospiraceae*, *Parabacteroides*, and *Faecalibacteium*	_	↓ TCA and T-DCA	↓ Body mass, fat mass, fat/lean index, liver weight; ↓ Liver TG	↓ Intestinal ceramide synthesis; ↑ GLP-1; Modulation of microbial BSH activity and inhibition of intestinal FXR signaling	Zhong et al. (2023)
↑ *Bacteroides*, *Ruminococcaceae*, and *Lachnospiraceae*	↑ ZO- 1, claudin-1, and occludin; ↓ CB1; ↑ CB2;	↓ LPS	↓ TG and blood glucose	↓ TNF-α, IL-1β and IL-6; ↑ IL-10; ↓ FAK, MyD88 and IRAK4	Chen et al. (2020a)
↓ *Firmicutes*/*Bacteroidetes* ratio; ↑ *Verrucomicrobia*; ↑ *Akkermansiaceae*; ↓ *Lachnospiraceae*, *Eubacterium*_*coprostanoligenes*_*group*, *Erysipelotrichaceae*, and *Peptostreptococcaceae*	↑ ZO- 1 and occludin	↑ BA circulation; ↓ Serum BA; ↑ Fecal TBA; ↓ LPS	↓ Body weight, liver weight, and epididymal fat weight; ↓ Serum TG, TC, LDL-C, ALT, AST; ↑ serum HDL-C	↓ TLR-4, NF-κB, and TNF-α; ↓ SREBP-1c and FAS; ↑ PPARα and CPT-1; ↑ FXR and CYP7A1	Duan et al. (2022)

**TABLE 2:** Polyherbal formulations for the treatment of MASLD.

Polyherbal formulations	Composition of the formula	Extraction	Metabolites identified by LC-MS	Study Type	Experiment Object (n)	Dosage and Intervention mode	Modeling methods
Zuogui Jiangtang Qinggan Fang	*Rehmannia glutinosa* (Gaertn.) Libosch. ex DC. [Orobanchaceae; Rehmanniae Radix Praeparata], *Astragalus membranaceus* Fisch. ex Bunge [Fabaceae; Astragali Radix], *Dioscorea polystachya* Turcz. [Dioscoreaceae; Dioscoreae Rhizoma], *Lycium chinense* Mill. [Solanaceae; Lycii Fructus], *Coptis chinensis* Franch. [Ranunculaceae; Coptidis Rhizoma], *Salvia miltiorrhiza* Bunge [Lamiaceae; Salviae Miltiorrhizae Radix et Rhizoma], *Artemisia capillaris* Thunb. [Asteraceae; Artemisiae Scopariae Herba], *Reynoutria japonica* Houtt. [Polygonaceae; Polygoni Cuspidati Rhizoma et Radix], *Curcuma rcenyujin* Y.H. Chenet C. Ling [Zingiberaceae; Curcumae Radix], *Citrus reticulata* Blanco [Rutaceae; Citri Reticulatae Pericarpium]	Formulated in the ratio 4:6:4:4:2:3:5:4:3:3, corresponding to: Rehmanniae Radix Praeparata (12 g), Astragali Radix (18 g), Dioscoreae Rhizoma (12 g), Lycii Fructus (12 g), Coptidis Rhizoma (6 g), Salviae Miltiorrhizae Radix et Rhizoma (9 g), Artemisiae Scopariae Herba (15 g), Polygoni Cuspidati Rhizoma et Radix (12 g), Curcumae Radix (9 g), Citri Reticulatae Pericarpium (9 g)	Rehmannioside D, Astragaloside IV, Calycosin-7-O-β-D-glucoside, Polysaccharides of Lycium chinense, Betaine, Scoparone, Emodin, Polydatin, Hesperidin, Nobiletin, etc.	*In vivo*	MKR transgenic mice, wild-type FVB inbred mice (40, 10 mice/group)	Low/High-dose ZGJTQGF: 7.5/15 g/kg, gavage 8 weeks	High-fat diet
Zexie-Baizhu Decoction	*Alisma orientalis* (Sam.) Juzep. [Alismataceae; Alismatis Rhizoma], *Aractylodes Macrocephala* Koidz [Asteraceae; Atractylodis Macrocephalae Rhizoma]	The components were mixed at 5:2 ratio, soaked in water (30 min) and decocted (2 h, low heat). The extract was vacuum-concentrated, lyophilized to powder (yield: 30%; storage: −20°C), and reconstituted in 0.5% sodium carboxymethyl cellulose (CMC-Na) before use	Monosaccharides and oligosaccharides 62.79%, Polysaccharides 19.26%, Amino acids 3.74%, Nucleosides 0.24%, Others 7.024%	*In vivo*	Male C57BL/6J mice (32, 8 mice/group)	1,500 mg/kg, oral administration 24 weeks	Gubra-Amylin NASH (GAN)-diet
Qushi Huayu formula	*Artemisia capillaris* Thunb. [Asteraceae; Artemisiae Scopariae Herba], *Reynoutria japonica* Houtt. [Polygonaceae; Polygoni Cuspidati Rhizoma et Radix], *Hypericum* Tourn. ex L. [Hypericaceae; Hyperici Perforati Herba], *Curcuma longa* L. [Zingiberaceae; Curcumae Longae Rhizoma], *Gardenia jasminoides* J.Ellis [Rubiaceae; Gardeniae Fructus]	QSHY granules were manufactured by Jiangyin Tianjiang Pharmaceutical Co., Ltd.	Quinic acid, Deacetylasperulosidic acid, Gallic acid, Neochlorogenicacid, Geniposidic acid, Shazhiside, 3,4-Dihydroxybenzaldehyde, Gardenoside, Picrocrocinic acid, etc.	*In vivo*	Patients with MASLD (246, 123/123)	QSHY granules (5.2 g, b.i.d.), oral administration 24 weeks	_
Qushi Huayu decoction	*Artemisia capillaris* Thunb. [Asteraceae; Artemisiae Scopariae Herba], *Reynoutria japonica* Houtt. [Polygonaceae; Polygoni Cuspidati Rhizoma et Radix], *Hypericum* Tourn. ex L. [Hypericaceae; Hyperici Perforati Herba], *Curcuma longa* L. [Zingiberaceae; Curcumae Longae Rhizoma], *Gardenia jasminoides* J.Ellis [Rubiaceae; Gardeniae Fructus]	Water extraction: Gardeniae fructus and Hyperici Perforati Herba underwent triple reflux extraction (1.5 h each) with 10× water (v/w). Post-concentration, the extract was ethanol-precipitated (60% v/v, 4°C, 12 h), followed by rotary evaporation (78°C, 220 Pa, 12 rpm). Ethanol extraction: Artemisiae Scopariae Herba, Polygoni Cuspidati Rhizoma et Radix, and Curcumae Longae Rhizoma were triple-extracted with 75% ethanol (1.5 h each). Ethanol was removed via identical evaporation parameters. *Final preparation:* Combined aqueous/ethanol extracts were condensed to 0.93 g crude herb/mL	Quinic acid, Deacetylasperulosidic acid, Gallic acid, Neochlorogenicacid, Geniposidic acid, Shazhiside, 3,4-Dihydroxybenzaldehyde, Gardenoside, Picrocrocinic acid, Chlorogenic acid, Caffeic acid, Genipin 1-gentiobioside, 4-Hydroxyacetophenone, Geniposide, Picrocrocin, Polydatin, etc.	*In vivo*	Male C57BL/6 mice (36, 9 mice/group)	0.93 g/kg, gavage 4 weeks	High-fat diet
Shenling Baizhu powder	*Panax ginseng* C. A. Mey. [Araliaceae; Ginseng Radix et Rhizoma], *Poria cocos* (Schw.) Wolf [Polyporaceae; Poria], *Aractylodes Macrocephala* Koidz [Asteraceae; Atractylodis Macrocephalae Rhizoma], *Dioscorea polystachya* Turcz. [Dioscoreaceae; Dioscoreae Rhizoma], *Lablab purpureus* (L.) Sweet [Fabaceae; Lablab Semen Album], *Nelumbo nucifera* Gaertn. [Nelumbonaceae; Nelumbinis Plumula], *Glycyrrhiza uralensis* Fisch. ex DC. [Fabaceae; Glycyrrhizae Radix Et Rhizoma], *Coix lacryma-jobi* L. [Poaceae; Coicis Semen], *Platycodon grandiflorus* (Jacq.) A.DC. [Campanulaceae; Platycodonis Radix], *Amomum villosum* Lour. [Zingiberaceae; Amomi Fructus]	Mix in a ratio of 5:5:5:5:4:3:3:3:2:2 in sequence. The formula granules were dissolved in distilled water and stored at −4 °C	Ginsenosides Rb, Ginsenosides Rc, Glycyrrhizinate, Atractylodes lactone Ⅲ, Atractylodes lactone Ⅱ, Atractylodes lactone Ⅰ, Poria aci, etc.	*In vivo*	Male Sprague-Dawley rats (72, 12 rats/group)	30 g/kg, gavage 16 weeks	High-fat diet
Shenling Baizhu powder	*Panax ginseng* C. A. Mey. [Araliaceae; Ginseng Radix et Rhizoma], *Poria cocos* (Schw.) Wolf [Polyporaceae; Poria], *Aractylodes Macrocephala* Koidz [Asteraceae; Atractylodis Macrocephalae Rhizoma], *Dioscorea polystachya* Turcz. [Dioscoreaceae; Dioscoreae Rhizoma], *Lablab purpureus* (L.) Sweet [Fabaceae; Lablab Semen Album], *Nelumbo nucifera* Gaertn. [Nelumbonaceae; Nelumbinis Plumula], *Glycyrrhiza uralensis* Fisch. ex DC. [Fabaceae; Glycyrrhizae Radix Et Rhizoma], *Coix lacryma-jobi* L. [Poaceae; Coicis Semen], *Platycodon grandiflorus* (Jacq.) A.DC. [Campanulaceae; Platycodonis Radix], *Amomum villosum* Lour. [Zingiberaceae; Amomi Fructus]	Prepare the SLBZP into an aqueous decoction according to the standard of taking orally at 6 g orally thrice daily, stored, sealed at 4°C and administered by heating in a water bath at 37°C during gavage	Quercetin, Quercetin-3-O-galactoside, Quercetin-3-O-galactoside, Ginsenoside Ro, Ginsenoside Rg3(S-FORM), Licoricesaponin G2, Ginsenoside Rg2, Atractylenolide III, Ginsenoside Rg5, etc.	*In vivo*	Male C57BL/6 mice (48, 8 mice/group)	Low/Middle/High-dose group: 2.34/4.68/9.36 g/kg, gavage 4 weeks	High-fat diet
Zhishi Daozhi Decoction	*Citrus aurantium* L. [Rutaceae; Aurantii Fructus], *Rheum palmatum* L. [Polygonaceae; Rhei Radix Et Rhizoma], *Coptis chinensis* Franch. [Ranunculaceae; Coptidis Rhizoma], *Scutellaria baicalensis* Georgi [Lamiaceae; Scutellariae Radix], *Monascus purpureus* Went. [Aspergillus; Fermentum Rubrum], *Aractylodes Macrocephala* Koidz [Asteraceae; Atractylodis Macrocephalae Rhizoma], *Poria cocos* (Schw.) Wolf [Polyporaceae; Poria], *Alisma orientalis* (Sam.) Juzep. [Alismataceae; Alismatis Rhizoma]	Herbal components [Aurantii Fructus (12.8 g), Rhei Radix Et Rhizoma (6.4 g), Coptidis Rhizoma (19.2 g), Scutellariae Radix (12.8 g), Fermentum Rubrum (19.2 g), Atractylodis Macrocephalae Rhizoma (19.2 g), Poria (19.2 g), Alismatis Rhizoma (12.8 g)] were decocted via two-stage extraction: Primary decoction: 300 mL H_2_O, 30 min; Secondary decoction: 200 mL H_2_O, 30 min. Combined filtrates were concentrated to 1.45 g/mL (crude drug equivalence) and stored at −4°C (ZDD preparation)	Synephrine, Anthraquinone, Berberine, Baicalin, Alisol B 23-acetate, Alisol C 23-acetate, etc.	*In vivo*	Male C57BL/6 mice (43, control group: 13 mice, 10 mice of the remaining three groups)	14.5 g/kg, gavage 4 weeks	High-fat diet
Si Miao Formula	*Phellodendron chinense* Schneid. [Rutaceae; Phellodendri Chinensis Cortex], *Atractylodes lancea* (Thunb.) DC. [Asteraceae; Atractylodis Rhizoma], *Coix lacryma-jobi* L. [Poaceae; Coicis Semen], *Achyranthes bidentata* Blume [Amaranthaceae; Achyranthis Bidentatae Radix]	Mix in a weight ratio proportion of 2:1:2:1 (Phellodendri Chinensis Cortex:Atractylodis Rhizoma:Coicis Semen:Achyranthis Bidentatae Radix) based on Chinese Pharmacopoeia 2020 edition	Ariginine, Betaine, Sucrose, Guanosine, Clansenamide, Magnoflorine, Phellodendrine, Neochlorogenic acid, Magnocurarine, Menisperine, Chlorogenic acid, 3-O-feruloylquinic acid, Cryptochlorogenic acid, etc.	*In vivo*	Male C57BL/6 mice	Low/High-SMF group: 10/20 g/kg, gavage 16 weeks	High fat/high sucrose diet
Lingguizhugan Decoction	*Poria cocos* (Schw.) Wolf [Polyporaceae; Poria], *Cinnamomum cassia* (L.) J. Presl [Lauraceae; Cinnamomi Ramulus], *Aractylodes Macrocephala* Koidz [Asteraceae; Atractylodis Macrocephalae Rhizoma], *Glycyrrhiza uralensis* Fisch. ex DC. [Fabaceae; Glycyrrhizae Radix et Rhizoma]	Extract the botanical drugs (4:3:3:2) twice at a ratio of 1:8 botanical drugs to water for 1.5 h each time	Cinnamaldehyde, Glycyrrhizic acid, 2-Atractylenolide, Pachymic acid	*In vivo*	Male mice	Low/Middle/High-dose group: 2.5/5/10 g/kg, gavage 4 weeks	High-fat diet
Chaihu Guizhi Ganjiang Decoction	*Bupleurum chinense* Franch. [Apiaceae; Bupleuri Radix], *Scutellaria baicalensis* Georgi [Lamiaceae; Scutellariae Radix], *Cinnamomum cassia* (L.) J.Presl [Lauraceae; Cinnamomi Ramulus], *Zingiber officinale* Roscoe [Zingiberaceae; Zingiberis Rhizoma], *Trichosanthes kirilowii* Maxim. [Cucurbitaceae; Trichosanthis Radix], *Ostrea Gigas* thunberg [Oyster; Ostreae Concha], *Glycyrrhiza uralensis* Fisch. ex DC. [Fabaceae; Glycyrrhizae Radix et Rhizoma]	In accordance with the compound proportion, the recommended amount of medicinal materials in CGGD was weighed and extracted twice with 10 water refluxes, 1 h each time, percolated with a 200-mesh filtration fabric, then merged with the column, evaporated, concentrated to a thick paste, and freeze-dried	Baicalin, Wogonoside, Glycyrrhizin, 6-shogaol, Wogonin, Saikosaponin A, Liquiritin apioside, Saikosaponin C, Liquiritin, 6-gingerol, Oroxylin A, Saikosaponin D, Isoliquiritin, Hispidulin, etc.	*In vivo*	Male Sprague-Dawley rats (40, 8 rats/group)	Low/Middle/High-dose group: 5.1/10.2/20.4 g/kg, gavage 6 weeks	MCD diet
Salvia-Nelumbinis naturalis formula	*Salvia miltiorrhiza* Bge. [Labiatae; Salviae Miltiorrhizae Radix et Rhizoma], *Semen Nelumbinis* [Nymphaeaceae; Nelumbinis Folium], *Artemisia capillaris* Thunb. [Asteraceae; Artemisiae Scopariae Herba], *Reynoutria japonica* Houtt. [Polygonaceae; Polygoni Cuspidati Rhizoma et Radix]	Mix in a ratio of 1.5:1:2.5:2.5:1.5 in sequence, and reflux extracted by water, which was subsequently concentrated, and extracted with ethanol	Tanshinone ⅡA, Danshensu, Salvianolic acid B, Nuciferine, Emodin, Chlorogenic acid, etc.	*In vivo*	Male C57BL/6 mice (24, 8 mice/group)	750 mg/kg, gavage 4 weeks	MCD diet
Qingrequzhuo capsule	*Morus alba* L. [Moraceae; Mori Folium], *Neopicrorhiza scrophulariiflora* (Pennell) D.Y.Hong [Plantaginaceae; Picrorhiaze Rhizoma], *Anemarrhena asphodeloides* Bunge [Asparagaceae; Anemarrhenae Rhizoma], *Plantago asiatica* L. [Plantaginaceae; Plantaginis Herba], *Citrus reticulata* Blanco [Rutaceae; Citri Reticulatae Pericarpium], *Carthamus tinctorius* L. [Asteraceae; Carthami Flos], *Rheum palmatum* L. [Polygonaceae; Rhei Radix Et Rhizoma], *Smilax glabra* Roxb. [Smilacaceae; Smilacis Glabrae Rhizoma], *Dioscorea polystachya* Turcz. [Dioscoreaceae; Dioscoreae Rhizoma], *Achyranthes bidentata* Blume [Amaranthaceae; Achyranthis Bidentatae Radix]	15 g of Mori Folium, 9 g of Picrorhiaze Rhizoma, 12 g of Anemarrhenae Rhizoma, 12 g of Plantaginis Herba, 15 g of Citri Reticulatae Pericarpium, 9 g of Carthami Flos, 6 g of Rhei Radix Et Rhizoma, 15 g of Smilacis Glabrae Rhizoma, 12 g of Dioscoreae Rhizoma, 12 g of Achyranthis Bidentatae Radix were weighed, mixed, decocted and evaporated to obtain the extract powder. The powders were made into capsule (0.5 g per capsule)	Rutin, Picroside Ⅰ, Picroside Ⅱ, Mangiferin, Timosaponin B Ⅱ, Plantamajoside, Astilbin, Hesperidin, Nobiletin, Safflomin A, Kaempferol, Rhaponiticin, Cyasterone, etc.	*In vivo*	Male C57BL/6 mice (60, 10 mice/group)	Low/Middle/High-dose group: 0.48/0.96/1.92 g/kg, gavage 6 weeks	Methionine and choline deficient (MCD) diet

Positive control group	Effects on gut microbiota	Effects on intestinal mucosal barrier	Gut microbiota-derived metabolites	Metabolic phenotypes	Mechanisms	References
Metformin: 0.067 g/kg, gavage 8 weeks	↓ *Firmicutes*, *Desulfobacterota*, and *Proteobacteria*; ↑ *Bacteroidota*; ↓ *Firmicutes*/*Bacteroidetes* ratio; ↓ *Lachnospiraceae* and *Desulfovibrionaceae*; ↑ *Muribaculaceae* and *Lactobacillaceae*; ↓ *Lachnospiraceae*_NK4A 136_group and *Desulfovibrio*; ↑ *Lactobacillus* and *Akkermansia*	↑ The ileum villus height, width and villus height/crypt depth ratio; ↓ Goblet cells; ↑ Claudin-1, ZO-1, and Occludin	↑ SCFA (acetate, propionate, butyrate, isobutyrate, valerate and isovalerate)	↓ Insulin resistance, liver weight, liver index, FBG, fasting insulin (FINS); ↓ Serum ALT, AST, TG, TC, and LDL-C	Activation of AMPK signaling; ↓ CD36, PPAR-γ, SREBP1-C, ACC, C/EBPα and LXR-α	Zou et al. (2023)
_	↓*Firmicutes*/*Bacteroidetes* ratio; ↑ *Akkermansia muciniphila*	Enhancement of intestinal barrier integrity	↑ SCFA (acetic acid and propionic acid); ↓ LCA/(LCA + CDCA) ratio and LCA/(LCA + UDCA) ratio	↓ Body weight, liver weight; ↓ ALT, AST, TG, TC, and LDL-C; ↓ Fasting blood glucose (FBG); ↑ Glucose tolerance; ↓ Lipid accumulation	↑ The BAs receptor *Gpbar1*; ↓The SCFAs receptor *FFAR2*	Shi et al. (2025)
_	↓*Firmicutes*/*Bacteroidetes* ratio; ↓ *Escherichia-Shigella*; ↑ *Bacteroides*, *Lachnoclostridium*, *Tyzzerella*, and *Butyricicoccus*	_	_	↓ Serum ALT; ↓ Liver fat	_	Liu et al. (2024)
Sodium butyrate (NaB): 200 mg/kg, gavage 4 weeks	↑ Parabacteroides; ↓ Odoribacter, Rikenella, Tyzzerella, Intestinibacter, Romboutsia and Lachnospiraceae	↑ ZO-1, and Occludin;	↓ LPS	↓ Body weight; ↓ Liver TG	↓ LBP, TLR4, CD14, p-NF-κB p65 subunit and p-IκB in liver tissue; ↓ TNF-α, IL-1β and IL-6; Inhibition of the MAPK pathway	Leng et al. (2020)
Probiotics: 0.6 g/kg, gavage 16 weeks	↓Firmicutes/Bacteroidetes ratio; ↑ *Actinobacteria* and *Cyanobacteria*; ↓ *Verrucomicrobium*; ↑ *Akkermansia*; ↓ *Blautia*, *Roseburia*, *Phascolarctobacterium*, and *Desulfovibrio*	Enhancement of intestinal barrier integrity	↓ LPS; ↑ SCFA	↓ Liver weight; ↓ Serum ALT, AST; ↓ Serum and liver TC, TG;	↓TLR4/MYD88 signaling; ↓ NLRP3 inflammasome	Zhang et al. (2018)
Metformin: 0.468 g/kg, gavage 4 weeks	↑ *Proteobacteria*, *Bacteroidetes*; ↓ *Firmicutes*; ↑ uncultured_bacterium_f_*Muribaculaceae*, *Lactobacillus,* and *Duchenne*; ↓ *Helicobacter*	_	_	↓ Weight, the liver-to-body ratio; ↓ AST and TBIL; ↓ Serum TC, TG, and LDL-C; ↑ Serum HDL-C	↓ UCP2; ↑ AMPK, IF1; regulation the UCP2/AMPK/IF1 signalling pathway, thereby affecting mitochondrial energy metabolism	Yao et al. (2023)
polyene phosphatidylcholine (PPC): 120 mg/kg, gavage 4 weeks	↓*Firmicutes*/*Bacteroidetes* ratio	↑ occludin and ZO-1	↑ SCFA (acetic acid, propionic acid and butyric acid)	↓ Weight; ↓ Serum TG, ALT, and AST	_	Bi et al. (2022)
_	↑ *Verrucomicrobia* and *Proteobacteria*; ↓ *Firmicutes*; ↑ *Akkermansia*, *Faecalibaculum*, and *Caproiciproducens*	↑ ZO-1	_	↓ Volume of adipocytes; Liver TG; ↓ Serum TC, LDL-C, ALT, AST; ↓ FBG, AUC values of both IPGTT, and IPITT; ↓ insulin resistance	↓ NLRP3, IL-1α, MCP-1	Han et al. (2021)
_	↑ *Bacteroidetes*; ↓ *Lactobacillus*; ↑ *Akkermansia*	_	↓ TαMCA, TβMCA; ↑ DCA	↓ ALT, AST, γ-GT, TG, TC, and LDL-C	↓ TNF-α, IL-6, and IL-1β; ↑ FXR, TGR5, and FGF15/19; ↓ SREBP-1c, CYP7A1, CYP8B1	Wang et al. (2024a)
_	↓ *Firmicutes*/*Bacteroidetes* ratio; ↑ *unclassified*_*f*_*Lachnospiraceae*, *Roseburia, Lactobacillus*, and *Lachnoclostridium*; ↓ *norank*_*f*_*Lachnospiraceae*, *norank*_*f*_*Oscillospiraceae*, *Allobaculum*, *NK4A214*_*group*, *norank*_*f*_*Erysipelotrichaceae*, etc.	↑ Goblet cell quantity, mucosal thickness, and muscle thickness; ↑ Claudin-1, ZO-1, and Occludin	_	↓ Liver index; ↓ Serum ALT and AST	↓ TNF-α, IL-6, and IL-1β; ↑ IL-4 and IL-10; ↑ SCFAs, FFAR2 and FFAR3; ↓ TLR4, MyD88 and phosphorylated NFκB p65; Inhibition of TLR4/MyD88/NFκB signaling pathway	Wu et al. (2024)
_	↓*Firmicutes*/*Bacteroidetes* ratio; ↓ *Micrococcaceae*, *Brevibacteriaceae*, *Dermabacteraceae*, *Clostridiaceae 1*, *Microbacteriaceae*, *Corynebacteriaceae* and *Atopobiaceae*; ↑ *Peptostreptococcaceae*, *Sphingomonadaceae*, *Prevotellaceae*, *Lachnospiraceae* and *Akkermansiaceae*	_	↓ Total BA; ↑ Fecal norDCA and norCA; ↓ GHCA, ωMCA, TωMCA, GCA and GDCA	↓ Liver TG and serum ALT	Activation of colonic FXR-FGF15 pathway	Li et al. (2021a)
polyene phosphatidylcholine (PPC): 88 mg/kg, gavage 6 weeks	↓*Firmicutes*/*Bacteroidetes* ratio; ↑ *Dubosiella* and *Blautia*; ↓ *Lachnospiraceae_NK4A136_group*	↑ ZO-1 and occludin	↓ LPS	↓ Hepatic lipid deposition	↓ TLR4, MyD88, and IκB and NF-κBp65 phosphorylation levels; ↓ The mRNA level of IL-1β, IL-6, and TNF-α; Inhibition of TLR4/NF-κB signaling pathway	Lv et al. (2022)

### 4.1 Botanical drugs improve MASLD by modulating gut microbiota

#### 4.1.1 Cassiae Semen

*Cassia obtusifolia L. (Fabaceae)* seeds, a botanical drug with dual medicinal and dietary applications, contain bioactive metabolites including anthraquinones, naphthalenes, and naphthalopyranones, which exhibit hepatoprotective, hypolipidemic, and hypoglycemic properties (Chen et al., 2023). Among these metabolites, aurantio-obtusin (AO) and rubrofusarin-6-β-gentiobioside (RG) are representative anthraquinone and naphthalopyranone derivatives. Chronic oral administration of AO (20 mg/kg/day for 16 weeks) in HFD-induced murine model restored intestinal barrier integrity and remodeled gut microbiota composition, notably reducing the *Firmicutes*/*Bacteroidetes* ratio while enriching beneficial taxa such as *Desulfovibrio* (genus), *Lachnoclostridium* (genus), *unclassified_f__Muribaculaceae* (genus), and *Lachnospiraceae_NK4A136_group* (genus). These microbial shifts correlated with enhanced SCFA production, improved hepatic lipid metabolism, and attenuated inflammation. AO further ameliorated hepatic steatosis by modulating PPAR signaling pathways and downregulating lipogenesis-associated genes (Li et al., 2025). In contrast, *Cassiae Semen* extract (CSE), a standardized hydroalcoholic extract of *Cassia obtusifolia* seeds, demonstrated broader therapeutic efficacy. CSE treatment significantly increased the abundance of commensal bacteria (*Dehalobacterium*, *Oscillospira*, *Ruminococcus*) while suppressing pathobionts (*Proteobacteria*, *Enterobacteriaceae*, *Erwinia*), effects linked to intestinal mucosal repair, reduced endotoxin levels, and diminished hepatic inflammation. FMT from CSE-treated mice into HFD-fed recipients replicated these benefits, restoring microbial diversity, enhancing barrier function, and mitigating metabolic and inflammatory liver injury (Luo et al., 2021). These findings demonstrate that the hepatoprotective effects of CSE are directly linked to its microbiota-modulating properties, providing direct evidence for the “microbiota-host” interaction mechanism.

Compared to the single metabolite AO, CSE exhibits broader regulatory capacity over gut microbiota, such as suppressing pathogenic taxa (e.g., *Proteobacteria*, *Erwinia*), highlighting the advantage of multi-component synergistic intervention. This aligns with the characteristic “multi-metabolite, multi-target” therapeutic strategy of TCM polyherbal formulations. However, several limitations should be noted. First, the specific molecular targets of AO in regulating PPAR signaling pathways (e.g., PPARα/γ/δ isoforms) and its potential epigenetic regulatory mechanisms remain unelucidated. Second, the contributions of other bioactive metabolites in CSE—beyond AO and RG—to microbiota modulation have not been individually dissected, making it challenging to distinguish the effects of single metabolites from those of the whole extract.

#### 4.1.2 Coptidis Rhizoma

Berberine (BBR), a bioactive isoquinoline alkaloid, is primarily isolated from botanical drugs including *Coptis chinensis* Franch. (Ranunculaceae), *Phellodendron chinense* C.K.Schneid. (Rutaceae), *Hydrastis canadensis* L. (Ranunculaceae), and *Berberis aquifolium* Pursh. (Berberidaceae). Preclinical studies have established its capacity to ameliorate MASLD through direct modulation of hepatic lipid metabolism (Yan et al., 2015). Mechanistic investigations by Shu et al. (Shu et al., 2021) further revealed that oral administration of BBR (100 mg/kg/day for 4 weeks) in HFD-fed C57BL/6J mice restored gut microbial homeostasis via selective enrichment of *Clostridiales* (order) and *Lactobacillaceae* (family), accompanied by expansion of *Bacteroidales* (order). These taxonomic shifts correlated with enhanced bile salt hydrolase (BSH) activity, facilitating the bioconversion of primary BAs to secondary forms such as deoxycholic acid (DCA) and ursodeoxycholic acid (UDCA). Functionally, DCA and UDCA act as agonists of the FXR, activating intestinal FXR signaling to upregulate FGF15 expression. This cascade ultimately attenuates hepatic steatosis and inflammation by suppressing *de novo* lipogenesis and NF-κB-mediated proinflammatory cytokine release (Wang G. et al., 2019; Zaufel et al., 2021).

By demonstrating that BBR-induced BA conversion directly activates intestinal FXR signaling, these study bridge microbial metabolic output (secondary BAs) to host pathophysiology, offering a mechanistic framework for microbiota-host crosstalk in MASLD. However, the specific contributions of *Clostridiales* versus *Lactobacillaceae* to BSH activity and BA bioconversion remain unresolved. Functional validation (e.g., BSH gene knockout strains) is needed to confirm taxonomic causality. While FXR activation is implicated, the roles of FXR isoforms (FXRα/β) are unaddressed.

#### 4.1.3 Puerariae Lobatae Radix

Puerarin, a bioactive isoflavone glycoside, is the principal metabolite derived from the roots of *Pueraria lobata* (Willd.) Ohwi (Fabaceae), a botanical drug traditionally used for its anti-inflammatory, antioxidant, and insulin-sensitizing properties (Zhou et al., 2014). Preclinical studies demonstrate its therapeutic potential in MASLD, with evidence highlighting its capacity to attenuate hepatic lipid accumulation, oxidative stress, and immune dysregulation (Xu et al., 2021; Zhou et al., 2022). In a methionine-choline-deficient (MCD) diet-induced murine model of MASH, oral administration of puerarin (200 mg/kg/day for 4 weeks) selectively suppressed the abundance of LPS-producing *Helicobacter* (genus) while enriching butyrate-generating *Roseburia* (genus), thereby ameliorating hepatic inflammation and steatosis (Gong et al., 2021). Butyrate, a SCFA critical for intestinal barrier integrity, mitigates endotoxemia by enhancing tight junction protein expression and suppressing NF-κB-driven proinflammatory cascades (Chen and Vitetta, 2020). Puerarin maintains gut homeostasis and the integrity of the intestinal mucosal barrier by enriching for the SCFA-producing bacteria *Lactobacillus*, *Bifidobacterium* and *Turicibacter*. In addition, these microbial shifts correlate with improved hepatic steatosis and lipid profiles (Yang et al., 2022).

These studies demonstrate that puerarin selectively suppresses the abundance of LPS-producing *Helicobacter* (genus) while enriching butyrate-generating *Roseburia* (genus). These microbial shifts are directly linked to butyrate-mediated intestinal barrier repair—evidenced by upregulated tight junction proteins—and inhibition of NF-κB-driven inflammatory signaling, thereby providing novel evidence for the “microbiota-host” interaction mechanism of isoflavonoids. However, the causal relationship between butyrate-induced NF-κB suppression and barrier restoration remains unelucidated, necessitating further validation through targeted metabolomics or functional assays using butyrate receptor antagonists (e.g., GPR109A).

#### 4.1.4 Ophiopogonis Radix

MDG-1, a water-soluble inulin-type β-D-fructan isolated from *Ophiopogon japonicus* (Thunb.) Ker Gawl. (Asparagaceae), demonstrates multifaceted therapeutic potential in MASLD. Previous studies established its capacity to ameliorate hepatic lipid accumulation, steatosis, and insulin resistance through PPAR signaling modulation (Wang et al., 2017). Further investigations revealed that MDG-1 alleviates HFD-induced metabolic disorders by normalizing BA metabolic pathways, particularly through FXR-mediated regulation of hepatic CYP7A1 and intestinal FGF15 expression (Shi et al., 2016). Notably, MDG-1 exerts systemic metabolic benefits via gut microbiota remodeling. Chronic administration of MDG-1 (8% w/w dietary supplementation for 8 weeks) in HFD-fed C57BL/6J mice restored microbial equilibrium, selectively enriching SCFA-producing taxa such as *Butyricimonas* (genus) and *Roseburia* (genus), while elevating fecal concentrations of acetic acid and valeric acid (Wang X. et al., 2019). These microbial shifts correlated with AMPK pathway activation, evidenced by upregulated AMPK, SREBP-1c, and ACC-1, ultimately rebalancing hepatic lipid synthesis and oxidation (Si et al., 2018). This suggests that MDG-1 may exert anti-MASLD effects by promoting the production of SCFA, which in turn activates the AMPK signaling pathway in the liver. However, the causal relationship between MDG-1-induced enrichment of *Butyricimonas*/*Roseburia* and AMPK pathway activation remains unelucidated. Functional validation through germ-free animal models or genetic knockout experiments targeting SCFA receptors (e.g., GPR43, GPR109A) is required to confirm the direct regulatory effects of microbial metabolites (e.g., acetate, valerate) on host signaling pathways.

#### 4.1.5 Dendrobii officinalis Caulis

*Dendrobium officinale* Kimura et Migo (Orchidaceae), a revered botanical drug in traditional medicine, contains bioactive metabolites including polysaccharides, phenanthrenes, and bibenzyls, which collectively exhibit hypolipidemic, hypoglycemic, hepatoprotective, and microbiota-modulating properties (Yang et al., 2020; Fang et al., 2022a). Mechanistic studies demonstrate its efficacy in countering lipid metabolic dysregulation. Polysaccharides isolated from *Dendrobium officinale* attenuate HFD-induced hepatic lipid deposition by ameliorating insulin resistance through PPAR-γ activation (Qu et al., 2021). Concurrently, standardized *D. officinale* extracts reduce oxidative stress and inflammatory cascades via inhibition of the NF-κB/IκB signaling axis, thereby mitigating hepatocyte injury (Zhou et al., 2025). Furthermore, *D. officinale* polysaccharides (250–1,000 mg/kg/day orally for 10 weeks) reshape gut microbiota composition in HFD-induced MASH rats, specifically suppressing intestinal barrier-disrupting taxa and LPS-producing bacteria. This microbiota remodeling prevents LPS translocation to the liver and inhibits TLR4/NF-κB pathway activation, resulting in attenuated hepatic inflammation and steatosis (Tian et al., 2023).

Despite significant advancements, critical limitations persist in current research: The specific molecular targets through which *D. officinale* polysaccharides regulate PPAR-γ or TLR4/NF-κB pathways—including receptor isoforms (e.g., PPAR-γ1/γ2) or epigenetic modifications (e.g., DNA methylation, histone acetylation)—remain unelucidated. Advanced techniques such as CRISPR-Cas9 knockout models or single-cell RNA sequencing are required to dissect these mechanisms. The chemical profile of *D. officinale* extracts—including polysaccharide molecular weight distribution and quantitative analysis of bibenzyls—fails to meet ConPhyMP guidelines for natural product quality control. Furthermore, synergistic effects between polysaccharides and other bioactive metabolites (e.g., phenanthrenes) remain unassessed, potentially underestimating the holistic therapeutic efficacy of the whole botanical drug.

#### 4.1.6 Astragali Radix

*Astragalus membranaceus* Fisch. ex Bunge (Fabaceae), a cornerstone botanical drug in TCM, produces bioactive metabolites including astragaloside IV (AS-IV) and *Astragalus* polysaccharides (APS), which exhibit hypoglycemic, hypolipidemic, and immunomodulatory properties (Fu et al., 2014). APS ameliorates HFD-induced metabolic dysfunction by remodeling gut microbiota composition and function (Hong et al., 2020). Specifically, APS administration (8% w/w dietary supplementation for 13 weeks) regulated serum and liver BA profiles in HFD-fed mice, especially increased serum taurohyodeoxycholic acid (THDCA) levels—a BA species associated with improved hepatic lipid deposition and glucose homeostasis (Zheng et al., 2024). However, the precise mechanism by which APS upregulates THDCA and its direct anti-steatotic effects remain uncharacterized, necessitating targeted metabolomic and receptor antagonist studies.

Further investigations revealed that APS alleviates MASLD in rats by rebalancing gut microbiota homeostasis, which in turn suppresses serum proinflammatory cytokines (e.g., TNF-α, IL-6) and activates the AMPK-PPAR-α signaling axis to inhibit *de novo* lipogenesis (Zhong et al., 2022). Parallel studies on AS-IV, a cycloartane-type triterpene glycoside, demonstrate its dual regulatory capacity: (1) suppression of TLR4/MyD88/NF-κB signaling, leading to downregulation of hepatic TNF-α, IL-6, and IL-8 in MASLD rats (Liu et al., 2020); (2) modulation of the FXR signaling pathway via gut microbiota-mediated BA metabolism. AS-IV reduces BSH activity, increasing intestinal tauro-β-muricholic acid (TβMCA), a potent FXR antagonist. This inhibits intestinal FXR-FGF15 signaling while activating hepatic FXR-SHP, collectively suppressing lipogenesis (Zhai et al., 2022). Notably, while TβMCA is murine-specific, its human analog glycoursodeoxycholic acid (GUDCA) shares similar FXR-modulating effects (Sun et al., 2018), underscoring the need for clinical trials to validate AS-IV’s translatability.

#### 4.1.7 Preliminary studies on additional botanical drugs

Emerging botanical drugs and their metabolites, though less extensively studied, demonstrate promising therapeutic potential for MASLD. Below, we critically evaluate their mechanisms of action through gut microbiota modulation.

Schisantherin A from *Schisandra chinensis* (Turcz.) Baill. (Schisandraceae): The lignan schisantherin A (80 mg/kg/day orally for 6 weeks) ameliorates hepatic steatosis in HFD-fed mice by restoring gut microbial diversity, particularly reducing the *Firmicutes*/*Bacteroidetes* ratio. This remodeling enhances intestinal barrier integrity (upregulated occludin) and reduces serum LPS levels, thereby inhibiting hepatic TLR4/NF-κB signaling and attenuating inflammation (Yu et al., 2022). However, the direct molecular targets linking schisantherin A to TLR4 pathway suppression (e.g., MyD88 phosphorylation or IRF3 activation) remain uncharacterized.

Caffeic Acid Phenethyl Ester (CAPE) from Chinese Propolis (*Populus* spp.): CAPE (75 mg/kg/day, 8 weeks), a major phenolic compound in Chinese propolis (15–29 mg/g) (Lv et al., 2021), mitigates HFD-induced MASLD by dual mechanisms: (1) inhibiting BSH activity, thereby increasing intestinal TCA and TDCA, which antagonizes FXR signaling; (2) reducing ceramide synthesis and enhancing GLP-1 secretion, collectively improving hepatic lipid metabolism (Zhong et al., 2023). Despite these insights, CAPE’s specificity for BSH inhibition (e.g., *Bacteroides* vs. *Clostridium* BSH isoforms) and its dose-dependent effects on FXR remain unaddressed.

Resveratrol from *Vitis vinifera* L. (Vitaceae): Resveratrol is a natural polyphenol that has been shown to be effective in improving liver inflammation and steatosis (Faghihzadeh et al., 2014; Faghihzadeh et al., 2015). Resveratrol (100 mg/kg/day, 12 weeks) attenuates MASLD progression via cannabinoid receptor modulation: inhibiting colonic CB1 receptors to preserve barrier function and activating CB2 receptors to suppress macrophage-driven inflammation (Chen M. et al., 2020). While effective in rodent models, its low oral bioavailability (<5%) and rapid phase II metabolism limit clinical utility. Nanoencapsulation strategies (e.g., chitosan-coated nanoparticles) may enhance its pharmacokinetic profile.

Tectorigenin (Tg) from *Belamcanda chinensis* (L.) Redouté (Iridaceae): Tg (25–50 mg/kg/day, 6 weeks), an isoflavone abundant in *Belamcanda* rhizomes, reshapes gut microbiota by promoting *Akkermansiaceae* (genus) and *Verrucomicrobia* (genus), which correlate with reduced hepatic TLR4/NF-κB activation and LPS-induced inflammation. Concurrently, Tg activates hepatic FXR and intestinal CYP7A1, accelerating BA synthesis and fecal excretion, thereby reducing lipotoxicity (Duan et al., 2022). However, the interplay between Tg-induced BA flux and microbiota remodeling requires validation using FXR knockout models.

In summary, preclinical studies highlight the therapeutic potential of botanical metabolites in MASLD through multi-target modulation of gut microbiota, including enrichment of SCFA-producing taxa, suppression of endotoxin-generating pathogens, and restoration of intestinal barrier integrity. These effects correlate with improved hepatic lipid metabolism and inflammation via pathways such as FXR/TLR4 signaling and AMPK activation. However, key limitations impede clinical translation. Firstly, overreliance on animal models with insufficient validation of bioavailability and *in vitro* efficacy. Secondly, incomplete elucidation of causal microbiota-host interactions and mechanistic signaling networks. Thirdly, lack of standardized phytochemical characterization and safety profiling for complex botanical drugs. Future research should prioritize translational strategies, including large-scale clinical trials with histological endpoints, advanced multi-omics integration to dissect metabolite-microbiota crosstalk, and nanotechnology-based delivery systems to enhance bioavailability. Additionally, mechanistic studies using germ-free models or receptor-specific knockouts are warranted to validate therapeutic targets and optimize botanical formulations for clinical application.

### 4.2 Polyherbal formulations improve MASLD by modulating gut microbiota

#### 4.2.1 Zuogui-Jiangtang-Qinggan-Fang

Zuogui-Jiangtang-Qinggan-Fang (ZGJTQGF), a decoction comprising ten botanical drugs including *A. membranaceus* Fisch. ex Bunge (Fabaceae), *C. chinensis* Franch. (Ranunculaceae), and *P. lobata* (Willd.) Ohwi (Fabaceae), demonstrates multi-target efficacy against MASLD. In HFD-induced C57BL/6J mice, oral administration of ZGJTQGF (15 g/kg for 8 weeks) attenuated hepatic steatosis by restoring lipid homeostasis and enhancing intestinal barrier integrity, as evidenced by upregulated tight junction proteins (ZO-1, occludin, and claudin-1). Gut microbiota analysis revealed significant enrichment of SCFA-producing taxa, including *Lactobacillaceae* (family), *Lactobacillus* (genus), *Akkermansia* (genus), and *Bacteroidota* (phylum), accompanied by elevated fecal acetate and butyrate concentrations (Zou et al., 2023). The synergistic effects of ZGJTQGF’s multi-component composition—such as berberine from *C. chinensis* (FXR activation) and puerarin from *P. lobata* (NF-κB inhibition)—highlight its “multi-metabolite, multi-pathway” therapeutic strategy. However, critical gaps persist: Firstly, the necessity of microbiota remodeling for ZGJTQGF’s efficacy remains unproven, requiring validation via FMT or germ-free models. Secondly, batch-to-batch variability in the decoction’s chemical profile (e.g., alkaloid/polysaccharide ratios) lacks standardization per ConPhyMP guidelines.

#### 4.2.2 Zexie-Baizhu Decoction

Zexie-Baizhu Decoction (AA), a Chinese classical formulation composed of *Alisma orientalis* (Sam.) Juzep. (Alismataceae) and *Atractylodes macrocephala* Koidz (Asteraceae) in a 5:2 ratio, has a long history of use in treating metabolic disorders. Multi-omics analysis showed that AA was able to regulate energy sensors, inhibit adipogenesis and alleviate lipid metabolism disorders (Cao Y. et al., 2022). In MASLD mice, AA administration (1.5 g/kg/day orally for 24 weeks) significantly ameliorated HFD-induced liver injury. Further analysis revealed that AA inhibited hepatic lipid deposition by regulating gut microbiota and its metabolites SCFA and BA, highlighting the potential of AA in regulating lipid metabolism (Shi et al., 2025). These findings provide new perspectives on the anti-MASLD effects of AA and deserve more in-depth studies to elucidate its specific mechanism.

#### 4.2.3 Qushi Huayu Formula

Qushi Huayu Formula (QSHY), a polyherbal preparation containing *Artemisia capillaris* Thunb. (Asteraceae), *Reynoutria japonica* Houtt. (Polygonaceae), *Hypericum japonicum* Thunb. ex Murray (Hypericaceae), *Curcuma longa* L. (Zingiberaceae), and *Gardenia jasminoides* Ellis. (Rubiaceae), demonstrates efficacy in improving hepatic lipid metabolism in MASLD patients (Liu et al., 2024). In HFD-induced murine model, QSHY (0.93 g/kg/day orally for 4 weeks) activated hepatic AMPK, downregulated sterol regulatory element-binding protein 1 (SREBP-1) and carbohydrate-responsive element-binding protein (ChREBP) expression, and suppressed *de novo* lipogenesis (Feng et al., 2013). Gut microbiota profiling revealed QSHY’s capacity to enrich *Parabacteroides* (genus). Additionally, QSHY inhibited colonic MAPK signaling, preserving tight junction integrity (ZO-1, occludin) and attenuating endotoxin translocation (Leng et al., 2020). However, the causal link between MAPK inhibition and microbiota remodeling requires validation via FMT studies.

#### 4.2.4 Shenling Baizhu Powder

Shenling Baizhu Powder (SLBZP), a spleen-invigorating formulation from the Song Dynasty’s *Taiping Huimin Hejiju Fang*, comprises 10 botanical drugs, including *Panax ginseng* C.A.Mey. (Araliaceae) and *Dioscorea polystachya* Turcz. (Dioscoreaceae). SLBZP (30 g/kg/day orally for 16 weeks) enhances gut microbiota-derived SCFAs by enriching *Bifidobacterium* (genus) and *Anaerostipes* (genus), which activate the UCP2/AMPK/IF1 signaling axis to boost hepatic mitochondrial ATP synthesis (Zhang et al., 2018; Yao et al., 2023). Parallel studies demonstrated that SLBZP reduces intestinal LPS translocation by suppressing TLR4/NLRP3 inflammasome activation, thereby downregulating hepatic pro-inflammatory factor expression (Pan et al., 2021). Nevertheless, the upstream regulators connecting TLR4 signaling to NLRP3 activation (e.g., MyD88/TRIF adaptors) remain uncharacterized.

#### 4.2.5 Zhishi Daozhi Decoction

Zhishi Daozhi Decoction (ZDD), a classical formulation comprising eight botanical drugs such as *Citrus aurantium* L. (Rutaceae), *Rheum palmatum* L. (Polygonaceae), *C. chinensis* Franch. (Ranunculaceae), and *Scutellaria baicalensis* Georgi (Lamiaceae), demonstrates therapeutic potential in MASLD. Preclinical studies in HFD-fed mice revealed that ZDD (14.5 g/kg/day orally for 4 weeks) modulates gut microbiota composition by decreasing *Firmicutes*/*Bacteroidetes* ratio, while enhancing intestinal barrier integrity via upregulation of occludin and ZO-1 (Bi et al., 2022). These changes correlated with reduced hepatic triglyceride content. However, the absence of FMT to validate causality and the lack of mechanistic details (e.g., specific microbial taxa or signaling pathways involved) limit the interpretability of these findings. Future studies should employ metagenomic sequencing and targeted metabolite profiling to dissect ZDD’s microbiota-dependent effects.

#### 4.2.6 Si Miao Formula

Si Miao Formula (SMF), a polyherbal preparation containing *P. chinense* C.K.Schneid. (Rutaceae) bark, *Atractylodes lancea* (Thunb.) DC. (Asteraceae) rhizome, *Coix lacryma-jobi* L. (Poaceae) seeds, and *Achyranthes bidentata* Blume (Amaranthaceae) roots, exhibits therapeutic effects on hyperuricemia and MASLD (Lin et al., 2020). Recent studies have found that SMF has the potential to ameliorate insulin resistance and inhibit adipogenesis (Jiang et al., 2022). In high fat/high sucrose diet-fed mice, SMF (10–20 g/kg for 16 weeks) ameliorated insulin resistance and hepatic lipid deposition by modulating gut microbiota (e.g., enriching *Akkermansia* [genus]). SMF suppressed hepatic *de novo* lipogenesis via FXR/SREBP-1c signaling inhibition, downregulating key lipogenic genes (*Acly*, *Fas*, *Acc*, and Scd-1) and pro-inflammatory cytokines (Han et al., 2021; Chen et al., 2022). Despite these benefits, the formulation’s batch-to-batch variability (e.g., alkaloid content from *P. chinense*) and the absence of clinical validation necessitate further standardization and human trials.

#### 4.2.7 Lingguizhugan Decoction

Lingguizhugan Decoction (LGZG), a four-herb formulation including *Poria cocos* (Schw.) Wolf (Polyporaceae), *Cinnamomum cassia* (L.) J. Presl (Lauraceae), *A. macrocephala* Koidz. (Asteraceae), and *Glycyrrhiza uralensis* Fisch. ex DC. (Fabaceae), improves insulin sensitivity and hepatic steatosis in MASLD (Dai et al., 2022). In HFD-induced MASH mice, LGZG (2.5–10 g/kg/day orally for 4 weeks) reshaped gut microbiota by increasing *Bacteroides* (genus) and *Akkermansia* (genus), while reducing tauro-α/β-muricholic acid (Tα/βMCA) levels and elevating deoxycholic acid (DCA). This BA profile activated hepatic FXR/TGR5 signaling, attenuating lipid accumulation (Wang J. et al., 2024). LGZG also inhibited the STING-TBK1-NF-κB pathway in Kupffer cells, mitigating LPS-induced oxidative stress and inflammation (Cao L. et al., 2022). Nevertheless, the interplay between microbiota remodeling and STING pathway suppression remains unelucidated, requiring co-culture models or single-cell transcriptomics for validation.

#### 4.2.8 Chaihu Guizhi Ganjiang Decoction

Chaihu Guizhi Ganjiang Decoction (CGGD), derived from *Treatise on Febrile Diseases*, contains seven botanical drugs, including *Bupleurum chinense* DC. (Apiaceae), *C. cassia* (L.). J.Presl (Lauraceae), and *S. baicalensis* Georgi (Lamiaceae). Numerous studies have shown that the metabolites of CGGD have various effects such as anti-inflammatory, antifibrotic and improved liver function (Ahn et al., 2021; Li X. et al., 2021). In MCD-induced MASH rats, CGGD (5.1–20.4 g/kg/day orally for 6 weeks) reduced pro-inflammatory taxa and preserved intestinal barrier function, thereby lowering hepatic LPS levels and TLR4/MyD88/NF-κB activation (Wu et al., 2024). Concurrently, CGGD enhanced PPARα-mediated fatty acid oxidation. While promising, the lack of chemical standardization (e.g., saikosaponin content from *B. chinense*) and clinical data limits its translational potential.

#### 4.2.9 Preliminary studies on additional polyherbal formulations

Salvia-Nelumbinis Naturalis (SNN) is a polyherbal formulation comprised of four botanical drugs, including *Salvia miltiorrhiza* Bge. (Lamiaceae), *Semen Nelumbinis*, *R. japonica* Houtt. (Polygonaceae), *A. capillaris* Thunb. (Asteraceae) (Ma et al., 2017). SNN (750 mg/kg/day orally for 4 weeks) attenuated LPS-induced intestinal barrier dysfunction by activating colonic FXR-FGF15 signaling and normalizing fecal BA profiles (Li C. et al., 2021). Qingrequzhuo capsule (QRQZ) improved hepatic inflammatory and lipid metabolism in MASH mice mainly by modulating gut microbiota and intestinal mucosal barrier, which in turn inhibits the TLR4/NF-κB pathway though its chemical composition remains uncharacterized (Lv et al., 2022). Yindanxinnaotong reduced hepatic lipid deposition via AMPK-mediated fatty acid oxidation, yet its multi-omics data lack functional validation (Huang et al., 2024).

In conclusion, polyherbal formulations demonstrate significant therapeutic potential for MASLD through microbiota-centric mechanisms. However, current studies on polyherbal formulations for MASLD exhibit the following shared limitations: Firstly, polyherbal formulations comprise chemically complex mixtures of botanical metabolites that interact with multiple host-microbiota targets. Current studies often fail to distinguish the contributions of individual components or validate causality via functional assays (e.g., FMT, germ-free models). Secondly, batch-to-batch variability in bioactive constituents (e.g., alkaloid content in SMF, saikosaponins in CGGD) undermining reproducibility. To address these gaps, future research must prioritize integrate multi-omics (metagenomics, metabolomics) with *in vitro* systems (gut-liver organoids) to deconvolute metabolite-microbiota-host interactions. Besides, establish standardized protocols for extract preparation (e.g., HPLC fingerprinting of ZGJTQGF’s berberine/puerarin ratios) and pharmacodynamic evaluation aligned with pharmacopeial standards.

## 5 Summary and prospects

MASLD remains a formidable clinical challenge, with current therapeutic strategies predominantly limited to lifestyle modifications and pharmacological agents offering marginal histological benefits. The absence of universally approved disease-modifying drugs underscores the urgent need for novel therapeutic paradigms. Emerging insights into the gut-liver axis have redefined MASLD as a multisystem disorder, where intestinal dysbiosis, mucosal barrier dysfunction, and microbiota-derived metabolites (e.g., LPS, SCFAs, BAs) drive hepatic inflammation and metabolic dysregulation. Clinical and preclinical evidence consistently links gut permeability alterations, endotoxemia, and microbial metabolite imbalances to MASLD progression, positioning intestinal homeostasis restoration as a pivotal therapeutic target. Current research on botanical drugs and metabolites interventions for MASLD highlights the following evidence-based advantages: 1. Multi-target regulation: Botanical drugs exert synergistic therapeutic effects by modulating gut microbiota composition (e.g., enriching SCFA-producing bacteria and suppressing endotoxin-generating taxa), restoring intestinal barrier integrity (via upregulation of TJ proteins), regulating BA metabolism (through FXR-FGF15 axis activation), and inhibiting inflammatory pathways (e.g., TLR4/NF-κB signaling). Representative plant-derived metabolites such as berberine, curcumin, and resveratrol, as well as standardized polyherbal formulations like Yinchenhao Decoction and Simiao Formula, exhibit systemic regulatory actions across these pathways. 2. Dual metabolic-immunological modulation: Botanical drugs not only ameliorate lipid dysregulation (e.g., SREBP1c inhibition and AMPK activation) but also attenuates hepatic injury by reshaping the intestinal immune microenvironment, including NLRP3 inflammasome suppression and Th1/Th2 balance restoration. This reflects a holistic therapeutic strategy centered on the gut-liver axis. 3. Enhanced safety and tolerability: Compared to conventional agents such as vitamin E (Chalasani et al., 2018; Powell et al., 2021) and pioglitazone (Tahara, 2021), botanical drugs and metabolites interventions (e.g., Spleen-Strengthening and Liver-Draining Formula, chitosan-coated curcumin) demonstrate lower risks of adverse effects in clinical trials, particularly suitable for long-term management. 4. Microbiota-dependent bioactivation: Gut microbial biotransformation of botanical drugs generates highly active metabolites (e.g., hydrolysis of flavonoid glycosides to aglycones), improving bioavailability and tissue specificity. This unique “host-microbe” collaborative mechanism underscores the pharmacological synergy between botanical drugs constituents and commensal microbiota.

Despite the remarkable progress made, we have to admit that there are still some key limitations in the current research on the treatment of MASLD with botanical drugs and metabolites: 1. Mechanistic superficiality: Most studies remain confined to correlational analyses, lacking causal validation of botanical metabolite-microbiota interactions or metabolite-host signaling pathways. Key gaps include insufficient use of genetically modified animal models (e.g., FXR or TLR4 knockouts) and germ-free systems to isolate microbial contributions. 2. Model inadequacy: Overreliance on homogeneous models—primarily HFD or MCD diet-induced male C57BL/6J mice—fails to recapitulate MASLD heterogeneity. Choline-deficient models rapidly induce MASH-fibrosis while showing relatively poor translatability. Conversely, HFD diet models mimic metabolic dysregulation but rarely develop significant fibrosis (Vacca et al., 2024). This “metabolism-fibrosis dichotomy” undermines pathological relevance. Additionally, female subjects and genetic diversity (e.g., ob/ob, db/db strains) remain underexplored. 3. Standardization deficits: Variability in botanical drug sourcing, extraction protocols (aqueous vs. ethanol), and polyherbal formulation ratios hinder cross-study reproducibility and global translation. 4. Clinical evidence limitations: Existing trials predominantly exhibit small cohorts (<100 participants), short durations (<6 months), and absence of liver biopsy-confirmed endpoints. Furthermore, personalized therapeutic responses linked to baseline microbiota signatures are rarely stratified. 5. Long-term safety uncertainty: Current clinical guidelines for MASLD lack specific recommendations for botanical drug applications, posing challenges in therapeutic implementation. Key limitations include insufficient characterization of long-term safety profiles and potential toxic metabolites. While berberine demonstrates metabolic benefits in MASLD with manageable gastrointestinal effects (e.g., nausea, diarrhea) (Nie et al., 2024), the toxicological risks of most phytochemicals remain unverified. Besides, pharmacological interactions between botanical and conventional drugs, where established combinations (e.g., warfarin, cyclosporine) may induce toxicity or reduce therapeutic efficacy (Awortwe et al., 2018). These knowledge gaps underscore the necessity for rigorous clinical evaluations of safety parameters and botanical drug-conventional drug interactions to enable standardized botanical therapy integration in MASLD management.

To address current limitations in TCM-based MASLD research, future studies must integrate cutting-edge technologies to achieve mechanistic and clinical breakthroughs. Firstly, Systematic integration of metagenomics, metabolomics, and proteomics can delineate gut microbiota signatures and metabolic networks modulated by botanical metabolites. AI algorithms (e.g., deep learning for network pharmacology) may predict tripartite interactions between plant-derived metabolites, microbial enzymes, and host targets, enabling rational optimization of polyherbal formulations and identification of bioactive metabolites (Niu et al., 2023; Qian et al., 2024). Secondly, leveraging rapidly advancing precision delivery technologies, engineered nanocarriers (e.g., lipid nanoparticles or exosomes) with colonic targeting capacity could enhance localized exposure of hydrophobic metabolites while minimizing systemic absorption. This approach may synergize with CRISPR-Cas9/phage-mediated ablation of pathobionts (e.g., ethanol-producing *K. pneumoniae*), enabling restoration of microbial ecology through pathogen-specific elimination without inducing broad-spectrum dysbiosis, particularly when combined with botanical drug interventions. Thirdly, botanical drug-based management of MASLD necessitates large-scale clinical validation and personalized therapeutic design. Multicenter RCTs with extended follow-up (>12 months) should adopt histological endpoints (NAS score, fibrosis stage) and stratify outcomes by baseline microbiota clusters. Machine learning-based integration of metagenomic, metabolomic, and host genomic data could guide precision prescriptions aligned with TCM syndrome patterns. Finally, proactive interdisciplinary exploration of therapeutic mechanisms is essential. Utilizing organoid models and single-cell sequencing technologies could unveil the multi-tissue regulatory effects of botanical drugs and polyherbal formulations on the intestinal epithelial-immune-hepatic stellate cell axis. In conclusion, the therapeutic potential of botanical drugs and metabolites in MASLD must be fully unlocked through technological innovation and interdisciplinary collaboration, thereby offering safer, more efficient systemic solutions for global MASLD management.

## References

[B1] Abdelmegeed M. A. Choi Y. Godlewski G. Ha S. K. Banerjee A. Jang S. (2017). Cytochrome P450-2E1 promotes fast food-mediated hepatic fibrosis. Sci. Rep. 7, 39764. 10.1038/srep39764 28051126 PMC5209674

[B2] Ahn J. Lee H. Jung C. H. Ha S. Y. Seo H. D. Kim Y. I. (2021). 6-Gingerol ameliorates hepatic steatosis via HNF4α/miR-467b-3p/GPAT1 cascade. Cell Mol. Gastroenterol. Hepatol. 12 (4), 1201–1213. 10.1016/j.jcmgh.2021.06.007 34139323 PMC8445893

[B3] Albillos A. de Gottardi A. Rescigno M. (2020). The gut-liver axis in liver disease: pathophysiological basis for therapy. J. Hepatol. 72 (3), 558–577. 10.1016/j.jhep.2019.10.003 31622696

[B4] Aljomah G. Baker S. S. Liu W. Kozielski R. Oluwole J. Lupu B. (2015). Induction of CYP2E1 in non-alcoholic fatty liver diseases. Exp. Mol. Pathol. 99 (3), 677–681. 10.1016/j.yexmp.2015.11.008 26551085 PMC4679539

[B5] An J. Liu Y. Wang Y. Fan R. Hu X. Zhang F. (2022). The role of intestinal mucosal barrier in autoimmune disease: a potential target. Front. Immunol. 13, 871713. 10.3389/fimmu.2022.871713 35844539 PMC9284064

[B6] Aoki R. Onuki M. Hattori K. Ito M. Yamada T. Kamikado K. (2021). Commensal microbe-derived acetate suppresses NAFLD/NASH development via hepatic FFAR2 signalling in mice. Microbiome 9 (1), 188. 10.1186/s40168-021-01125-7 34530928 PMC8447789

[B7] Awortwe C. Makiwane M. Reuter H. Muller C. Louw J. Rosenkranz B. (2018). Critical evaluation of causality assessment of herb-drug interactions in patients. Br. J. Clin. Pharmacol. 84 (4), 679–693. 10.1111/bcp.13490 29363155 PMC5867089

[B8] Baumann A. Jin C. J. Brandt A. Sellmann C. Nier A. Burkard M. (2020). Oral supplementation of sodium butyrate attenuates the progression of non-alcoholic steatohepatitis. Nutrients 12 (4), 951. 10.3390/nu12040951 32235497 PMC7231312

[B9] Bi C. R. Sun J. T. Du J. Chu L. Y. Li Y. J. Jia X. Y. (2022). Effects of Zhishi Daozhi Decoction on the intestinal flora of nonalcoholic fatty liver disease mice induced by a high-fat diet. Front. Cell Infect. Microbiol. 12, 1005318. 10.3389/fcimb.2022.1005318 36683694 PMC9846642

[B10] Buzzetti E. Pinzani M. Tsochatzis E. A. (2016). The multiple-hit pathogenesis of non-alcoholic fatty liver disease (NAFLD). Metabolism 65 (8), 1038–1048. 10.1016/j.metabol.2015.12.012 26823198

[B11] Cao L. Xu E. Zheng R. Zhangchen Z. Zhong R. Huang F. (2022a). Traditional Chinese medicine Lingguizhugan decoction ameliorate HFD-induced hepatic-lipid deposition in mice by inhibiting STING-mediated inflammation in macrophages. Chin. Med. 17 (1), 7. 10.1186/s13020-021-00559-3 34983596 PMC8728979

[B12] Cao Y. Shi J. Song L. Xu J. Lu H. Sun J. (2022b). Multi-omics integration analysis identifies lipid disorder of a non-alcoholic fatty liver disease (NAFLD) mouse model improved by zexie-baizhu decoction. Front. Pharmacol. 13, 858795. 10.3389/fphar.2022.858795 35795562 PMC9251488

[B13] Carpi R. Z. Barbalho S. M. Sloan K. P. Laurindo L. F. Gonzaga H. F. Grippa P. C. (2022). The effects of probiotics, prebiotics and synbiotics in non-alcoholic fat liver disease (nafld) and non-alcoholic steatohepatitis (nash): a systematic review. Int. J. Mol. Sci. 23 (15), 8805. 10.3390/ijms23158805 35955942 PMC9369010

[B14] Chalasani N. Younossi Z. Lavine J. E. Charlton M. Cusi K. Rinella M. (2018). The diagnosis and management of nonalcoholic fatty liver disease: practice guidance from the American Association for the Study of Liver Diseases. Hepatology 67 (1), 328–357. 10.1002/hep.29367 28714183

[B15] Chen J. Vitetta L. (2020). Gut microbiota metabolites in NAFLD pathogenesis and therapeutic implications. Int. J. Mol. Sci. 21 (15), 5214. 10.3390/ijms21155214 32717871 PMC7432372

[B16] Chen M. Hou P. Zhou M. Ren Q. Wang X. Huang L. (2020a). Resveratrol attenuates high-fat diet-induced non-alcoholic steatohepatitis by maintaining gut barrier integrity and inhibiting gut inflammation through regulation of the endocannabinoid system. Clin. Nutr. 39 (4), 1264–1275. 10.1016/j.clnu.2019.05.020 31189495

[B17] Chen X. Zhang Z. Li H. Zhao J. Wei X. Lin W. (2020b). Endogenous ethanol produced by intestinal bacteria induces mitochondrial dysfunction in non-alcoholic fatty liver disease. J. Gastroenterol. Hepatol. 35 (11), 2009–2019. 10.1111/jgh.15027 32150306

[B18] Chen Y. Chen X. Yang X. Gao P. Yue C. Wang L. (2023). Cassiae Semen: a comprehensive review of botany, traditional use, phytochemistry, pharmacology, toxicity, and quality control. J. Ethnopharmacol. 306, 116199. 10.1016/j.jep.2023.116199 36702448

[B19] Chen Y. Zhu L. Hu W. Wang Y. Wen X. Yang J. (2022). Simiao Wan modulates the gut microbiota and bile acid metabolism during improving type 2 diabetes mellitus in mice. Phytomedicine 104, 154264. 10.1016/j.phymed.2022.154264 35752076

[B20] Chopyk D. M. Grakoui A. (2020). Contribution of the intestinal microbiome and gut barrier to hepatic disorders. Gastroenterology 159 (3), 849–863. 10.1053/j.gastro.2020.04.077 32569766 PMC7502510

[B21] Cornejo-Pareja I. Amiar M. R. Ocana-Wilhelmi L. Soler-Humanes R. Arranz-Salas I. Garrido-Sanchez L. (2024). Non-alcoholic fatty liver disease in patients with morbid obesity: the gut microbiota axis as a potential pathophysiology mechanism. J. Gastroenterol. 59 (4), 329–341. 10.1007/s00535-023-02075-7 38265508 PMC10959783

[B22] Craven L. Rahman A. Nair Parvathy S. Beaton M. Silverman J. Qumosani K. (2020). Allogenic fecal microbiota transplantation in patients with nonalcoholic fatty liver disease improves abnormal small intestinal permeability: a randomized control trial. Am. J. Gastroenterol. 115 (7), 1055–1065. 10.14309/ajg.0000000000000661 32618656

[B23] Dai L. Xu J. Liu B. Dang Y. Wang R. Zhuang L. (2022). Lingguizhugan Decoction, a Chinese herbal formula, improves insulin resistance in overweight/obese subjects with non-alcoholic fatty liver disease: a translational approach. Front. Med. 16 (5), 745–759. 10.1007/s11684-021-0880-3 35471471

[B24] Dai X. Hou H. Zhang W. Liu T. Li Y. Wang S. (2020). Microbial metabolites: critical regulators in NAFLD. Front. Microbiol. 11, 567654. 10.3389/fmicb.2020.567654 33117316 PMC7575719

[B25] De A. Bhagat N. Mehta M. Taneja S. Duseja A. (2024). Metabolic dysfunction-associated steatotic liver disease (MASLD) definition is better than MAFLD criteria for lean patients with NAFLD. J. Hepatol. 80 (2), e61–e62. 10.1016/j.jhep.2023.07.031 37558135

[B26] Deng Y. Hu M. Huang S. Fu N. (2024). Molecular mechanism and therapeutic significance of essential amino acids in metabolically associated fatty liver disease. J. Nutr. Biochem. 126, 109581. 10.1016/j.jnutbio.2024.109581 38219809

[B27] Duan R. Huang K. Guan X. Li S. Xia J. Shen M. (2022). Tectorigenin ameliorated high-fat diet-induced nonalcoholic fatty liver disease through anti-inflammation and modulating gut microbiota in mice. Food Chem. Toxicol. 164, 112948. 10.1016/j.fct.2022.112948 35390440

[B28] Dunagan M. Chaudhry K. Samak G. Rao R. K. (2012). Acetaldehyde disrupts tight junctions in Caco-2 cell monolayers by a protein phosphatase 2A-dependent mechanism. Am. J. Physiol. Gastrointest. Liver Physiol. 303 (12), G1356–G1364. 10.1152/ajpgi.00526.2011 23064762 PMC4073985

[B29] Ekstrand M. I. Terzioglu M. Galter D. Zhu S. Hofstetter C. Lindqvist E. (2007). Progressive parkinsonism in mice with respiratory-chain-deficient dopamine neurons. Proc. Natl. Acad. Sci. U. S. A. 104 (4), 1325–1330. 10.1073/pnas.0605208103 17227870 PMC1783140

[B30] Estes C. Anstee Q. M. Arias-Loste M. T. Bantel H. Bellentani S. Caballeria J. (2018). Modeling NAFLD disease burden in China, France, Germany, Italy, Japan, Spain, United Kingdom, and United States for the period 2016-2030. J. Hepatol. 69 (4), 896–904. 10.1016/j.jhep.2018.05.036 29886156

[B31] Faderl M. Noti M. Corazza N. Mueller C. (2015). Keeping bugs in check: the mucus layer as a critical component in maintaining intestinal homeostasis. IUBMB Life 67 (4), 275–285. 10.1002/iub.1374 25914114

[B32] Faghihzadeh F. Adibi P. Hekmatdoost A. (2015). The effects of resveratrol supplementation on cardiovascular risk factors in patients with non-alcoholic fatty liver disease: a randomised, double-blind, placebo-controlled study. Br. J. Nutr. 114 (5), 796–803. 10.1017/S0007114515002433 26234526

[B33] Faghihzadeh F. Adibi P. Rafiei R. Hekmatdoost A. (2014). Resveratrol supplementation improves inflammatory biomarkers in patients with nonalcoholic fatty liver disease. Nutr. Res. 34 (10), 837–843. 10.1016/j.nutres.2014.09.005 25311610

[B34] Fang J. Lin Y. Xie H. Farag M. A. Feng S. Li J. (2022a). Dendrobium officinale leaf polysaccharides ameliorated hyperglycemia and promoted gut bacterial associated SCFAs to alleviate type 2 diabetes in adult mice. Food Chem. X 13, 100207. 10.1016/j.fochx.2022.100207 35498995 PMC9039915

[B35] Fang J. Yu C. H. Li X. J. Yao J. M. Fang Z. Y. Yoon S. H. (2022b). Gut dysbiosis in nonalcoholic fatty liver disease: pathogenesis, diagnosis, and therapeutic implications. Front. Cell Infect. Microbiol. 12, 997018. 10.3389/fcimb.2022.997018 36425787 PMC9679376

[B36] Feng Q. Gou X. J. Meng S. X. Huang C. Zhang Y. Q. Tang Y. J. (2013). Qushi Huayu decoction inhibits hepatic lipid accumulation by activating AMP-activated protein kinase *in vivo* and *in vitro* . Evid. Based Complement. Altern. Med. 2013, 184358. 10.1155/2013/184358 PMC3614185 23573117

[B37] Ferro D. Baratta F. Pastori D. Cocomello N. Colantoni A. Angelico F. (2020). New insights into the pathogenesis of non-alcoholic fatty liver disease: gut-derived lipopolysaccharides and oxidative stress. Nutrients 12 (9), 2762. 10.3390/nu12092762 32927776 PMC7551294

[B38] Frost F. Kacprowski T. Ruhlemann M. Pietzner M. Bang C. Franke A. (2021). Long-term instability of the intestinal microbiome is associated with metabolic liver disease, low microbiota diversity, diabetes mellitus and impaired exocrine pancreatic function. Gut 70 (3), 522–530. 10.1136/gutjnl-2020-322753 33168600 PMC7873430

[B39] Fu J. Wang Z. Huang L. Zheng S. Wang D. Chen S. (2014). Review of the botanical characteristics, phytochemistry, and pharmacology of Astragalus membranaceus (Huangqi). Phytother. Res. 28 (9), 1275–1283. 10.1002/ptr.5188 25087616

[B40] Gan L. Feng Y. Du B. Fu H. Tian Z. Xue G. (2023). Bacteriophage targeting microbiota alleviates non-alcoholic fatty liver disease induced by high alcohol-producing *Klebsiella pneumoniae* . Nat. Commun. 14 (1), 3215. 10.1038/s41467-023-39028-w 37270557 PMC10239455

[B41] Giorgio V. Miele L. Principessa L. Ferretti F. Villa M. P. Negro V. (2014). Intestinal permeability is increased in children with non-alcoholic fatty liver disease, and correlates with liver disease severity. Dig. Liver Dis. 46 (6), 556–560. 10.1016/j.dld.2014.02.010 24631029

[B42] Gong M. J. Zhu C. Y. Zou Z. J. Han B. Huang P. (2021). Therapeutic potential of puerarin against methionine-choline-deficient diet-induced non-alcoholic steatohepatitis determined by combination of (1)H NMR spectroscopy-based metabonomics and 16S rRNA gene sequencing. J. Pharm. Biomed. Anal. 197, 113964. 10.1016/j.jpba.2021.113964 33601157

[B43] Guo S. Al-Sadi R. Said H. M. Ma T. Y. (2013). Lipopolysaccharide causes an increase in intestinal tight junction permeability *in vitro* and *in vivo* by inducing enterocyte membrane expression and localization of TLR-4 and CD14. Am. J. Pathol. 182 (2), 375–387. 10.1016/j.ajpath.2012.10.014 23201091 PMC3562736

[B44] Gupta B. Liu Y. Chopyk D. M. Rai R. P. Desai C. Kumar P. (2020). Western diet-induced increase in colonic bile acids compromises epithelial barrier in nonalcoholic steatohepatitis. FASEB J. 34 (5), 7089–7102. 10.1096/fj.201902687R 32275114 PMC7831197

[B45] Han R. Qiu H. Zhong J. Zheng N. Li B. Hong Y. (2021). Si Miao Formula attenuates non-alcoholic fatty liver disease by modulating hepatic lipid metabolism and gut microbiota. Phytomedicine 85, 153544. 10.1016/j.phymed.2021.153544 33773192

[B46] Hasegawa T. Iino C. Endo T. Mikami K. Kimura M. Sawada N. (2020). Changed amino acids in nafld and liver fibrosis: a large cross-sectional study without influence of insulin resistance. Nutrients 12 (5), 1450. 10.3390/nu12051450 32429590 PMC7284573

[B47] Hong Y. Li B. Zheng N. Wu G. Ma J. Tao X. (2020). Integrated metagenomic and metabolomic analyses of the effect of Astragalus polysaccharides on alleviating high-fat diet-induced metabolic disorders. Front. Pharmacol. 11, 833. 10.3389/fphar.2020.00833 32587515 PMC7299173

[B48] Hrncir T. Hrncirova L. Kverka M. Hromadka R. Machova V. Trckova E. (2021). Gut microbiota and NAFLD: pathogenetic mechanisms, microbiota signatures, and therapeutic interventions. Microorganisms 9 (5), 957. 10.3390/microorganisms9050957 33946843 PMC8146698

[B49] Huang L. Rao Q. Wang C. Mou Y. Zheng X. Hu E. (2024). Multi-omics joint analysis reveals that the Miao medicine Yindanxinnaotong formula attenuates non-alcoholic fatty liver disease. Phytomedicine 135, 156026. 10.1016/j.phymed.2024.156026 39388921

[B50] Huang Z. R. Deng J. C. Li Q. Y. Cao Y. J. Lin Y. C. Bai W. D. (2020). Protective mechanism of common buckwheat (fagopyrum esculentum moench.) against nonalcoholic fatty liver disease associated with dyslipidemia in mice fed a high-fat and high-cholesterol diet. J. Agric. Food Chem. 68 (24), 6530–6543. 10.1021/acs.jafc.9b08211 32383865

[B51] Inagaki T. Moschetta A. Lee Y. K. Peng L. Zhao G. Downes M. (2006). Regulation of antibacterial defense in the small intestine by the nuclear bile acid receptor. Proc. Natl. Acad. Sci. U. S. A. 103 (10), 3920–3925. 10.1073/pnas.0509592103 16473946 PMC1450165

[B52] Jacob J. S. Ahmed A. Cholankeril G. (2021). The impact of alteration in gut microbiome in the pathogenesis of nonalcoholic fatty liver disease. Curr. Opin. Infect. Dis. 34 (5), 477–482. 10.1097/QCO.0000000000000759 34267042

[B53] Ji Y. Yin Y. Li Z. Zhang W. (2019). Gut microbiota-derived components and metabolites in the progression of non-alcoholic fatty liver disease (NAFLD). Nutrients 11 (8), 1712. 10.3390/nu11081712 31349604 PMC6724003

[B54] Jiang Q. X. Chen Y. M. Ma J. J. Wang Y. P. Li P. Wen X. D. (2022). Effective fraction from Simiao Wan prevents hepatic insulin resistant by inhibition of lipolysis via AMPK activation. Chin. J. Nat. Med. 20 (3), 161–176. 10.1016/S1875-5364(21)60115-2 35369960

[B55] Jiao N. Baker S. S. Chapa-Rodriguez A. Liu W. Nugent C. A. Tsompana M. (2018). Suppressed hepatic bile acid signalling despite elevated production of primary and secondary bile acids in NAFLD. Gut 67 (10), 1881–1891. 10.1136/gutjnl-2017-314307 28774887

[B56] Kayama H. Okumura R. Takeda K. (2020). Interaction between the microbiota, epithelia, and immune cells in the intestine. Annu. Rev. Immunol. 38, 23–48. 10.1146/annurev-immunol-070119-115104 32340570

[B57] Lang S. Demir M. Martin A. Jiang L. Zhang X. Duan Y. (2020). Intestinal virome signature associated with severity of nonalcoholic fatty liver disease. Gastroenterology 159 (5), 1839–1852. 10.1053/j.gastro.2020.07.005 32652145 PMC8404510

[B58] Le M. H. Yeo Y. H. Zou B. Barnet S. Henry L. Cheung R. (2022). Forecasted 2040 global prevalence of nonalcoholic fatty liver disease using hierarchical bayesian approach. Clin. Mol. Hepatol. 28 (4), 841–850. 10.3350/cmh.2022.0239 36117442 PMC9597215

[B59] Lena I. Parrot S. Deschaux O. Muffat-Joly S. Sauvinet V. Renaud B. (2005). Variations in extracellular levels of dopamine, noradrenaline, glutamate, and aspartate across the sleep--wake cycle in the medial prefrontal cortex and nucleus accumbens of freely moving rats. J. Neurosci. Res. 81 (6), 891–899. 10.1002/jnr.20602 16041801

[B60] Leng J. Huang F. Hai Y. Tian H. Liu W. Fang Y. (2020). Amelioration of non-alcoholic steatohepatitis by Qushi Huayu decoction is associated with inhibition of the intestinal mitogen-activated protein kinase pathway. Phytomedicine 66, 153135. 10.1016/j.phymed.2019.153135 31790895

[B61] Leon-Mimila P. Villamil-Ramirez H. Li X. S. Shih D. M. Hui S. T. Ocampo-Medina E. (2021). Trimethylamine N-oxide levels are associated with NASH in obese subjects with type 2 diabetes. Diabetes Metab. 47 (2), 101183. 10.1016/j.diabet.2020.07.010 32791310 PMC8018562

[B62] Li C. Zhou W. Li M. Shu X. Zhang L. Ji G. (2021a). Salvia-Nelumbinis naturalis extract protects mice against MCD diet-induced steatohepatitis via activation of colonic FXR-FGF15 pathway. Biomed. Pharmacother. 139, 111587. 10.1016/j.biopha.2021.111587 33865013

[B63] Li X. Ge J. Li Y. Cai Y. Zheng Q. Huang N. (2021b). Integrative lipidomic and transcriptomic study unravels the therapeutic effects of saikosaponins A and D on non-alcoholic fatty liver disease. Acta Pharm. Sin. B 11 (11), 3527–3541. 10.1016/j.apsb.2021.03.018 34900534 PMC8642447

[B64] Li Z. Jin Y. Zhao H. Gu Y. Zhang Y. Cheng S. (2025). Aurantio-obtusin regulates gut microbiota and serum metabolism to alleviate high-fat diet-induced obesity-associated non-alcoholic fatty liver disease in mice. Phytother. Res. 39, 1946–1965. 10.1002/ptr.8459 39953693

[B65] Lin X. Shao T. Huang L. Wen X. Wang M. Wen C. (2020). Simiao decoction alleviates gouty arthritis by modulating proinflammatory cytokines and the gut ecosystem. Front. Pharmacol. 11, 955. 10.3389/fphar.2020.00955 32670069 PMC7327538

[B66] Liu B. Qian J. Wang Q. Wang F. Ma Z. Qiao Y. (2014). Butyrate protects rat liver against total hepatic ischemia reperfusion injury with bowel congestion. PLoS One 9 (8), e106184. 10.1371/journal.pone.0106184 25171217 PMC4149529

[B67] Liu J. Wu A. Cai J. She Z. G. Li H. (2022). The contribution of the gut-liver axis to the immune signaling pathway of NAFLD. Front. Immunol. 13, 968799. 10.3389/fimmu.2022.968799 36119048 PMC9471422

[B68] Liu Q. Li X. Pan Y. Liu Q. Li Y. He C. (2024). Efficacy and safety of Qushi Huayu, a traditional Chinese medicine, in patients with nonalcoholic fatty liver disease in a randomized controlled trial. Phytomedicine 130, 155398. 10.1016/j.phymed.2024.155398 38788390

[B69] Liu W. Baker R. D. Bhatia T. Zhu L. Baker S. S. (2016). Pathogenesis of nonalcoholic steatohepatitis. Cell Mol. Life Sci. 73 (10), 1969–1987. 10.1007/s00018-016-2161-x 26894897 PMC11108381

[B70] Liu Y. L. Zhang Q. Z. Wang Y. R. Fu L. N. Han J. S. Zhang J. (2020). Astragaloside IV improves high-fat diet-induced hepatic steatosis in nonalcoholic fatty liver disease rats by regulating inflammatory factors level via TLR4/NF-κB signaling pathway. Front. Pharmacol. 11, 605064. 10.3389/fphar.2020.605064 33708118 PMC7941269

[B71] Loomba R. Seguritan V. Li W. Long T. Klitgord N. Bhatt A. (2017). Gut microbiome-based metagenomic signature for non-invasive detection of advanced fibrosis in human nonalcoholic fatty liver disease. Cell Metab. 25 (5), 1054–1062. 10.1016/j.cmet.2017.04.001 28467925 PMC5502730

[B72] Luo H. Wu H. Wang L. Xiao S. Lu Y. Liu C. (2021). Hepatoprotective effects of Cassiae Semen on mice with non-alcoholic fatty liver disease based on gut microbiota. Commun. Biol. 4 (1), 1357. 10.1038/s42003-021-02883-8 34862475 PMC8642482

[B73] Lv L. Cui H. Ma Z. Liu X. Yang L. (2021). Recent progresses in the pharmacological activities of caffeic acid phenethyl ester. Naunyn Schmiedeb. Arch. Pharmacol. 394 (7), 1327–1339. 10.1007/s00210-021-02054-w 33492405

[B74] Lv S. Zhang Z. Su X. Li W. Wang X. Pan B. (2022). Qingrequzhuo capsule alleviated methionine and choline deficient diet-induced nonalcoholic steatohepatitis in mice through regulating gut microbiota, enhancing gut tight junction and inhibiting the activation of TLR4/NF-κB signaling pathway. Front. Endocrinol. (Lausanne) 13, 1106875. 10.3389/fendo.2022.1106875 36743916 PMC9892721

[B75] Ma Z. Shu X. Huang J. Zhang H. Xiao Z. Zhang L. (2017). Salvia-nelumbinis naturalis formula improved inflammation in LPS stressed macrophages via upregulating MicroRNA-152. Mediat. Inflamm. 2017, 5842747. 10.1155/2017/5842747 PMC5266850 28167852

[B76] Macia L. Tan J. Vieira A. T. Leach K. Stanley D. Luong S. (2015). Metabolite-sensing receptors GPR43 and GPR109A facilitate dietary fibre-induced gut homeostasis through regulation of the inflammasome. Nat. Commun. 6, 6734. 10.1038/ncomms7734 25828455

[B77] Matthews D. R. Li H. Zhou J. Li Q. Glaser S. Francis H. (2021). Methionine- and choline-deficient diet-induced nonalcoholic steatohepatitis is associated with increased intestinal inflammation. Am. J. Pathol. 191 (10), 1743–1753. 10.1016/j.ajpath.2021.06.010 34242656 PMC8485057

[B78] McGlinchey A. J. Govaere O. Geng D. Ratziu V. Allison M. Bousier J. (2022). Metabolic signatures across the full spectrum of non-alcoholic fatty liver disease. JHEP Rep. 4 (5), 100477. 10.1016/j.jhepr.2022.100477 35434590 PMC9006858

[B79] Merlen G. Kahale N. Ursic-Bedoya J. Bidault-Jourdainne V. Simerabet H. Doignon I. (2020). TGR5-dependent hepatoprotection through the regulation of biliary epithelium barrier function. Gut 69 (1), 146–157. 10.1136/gutjnl-2018-316975 30723104

[B80] Misrani A. Tabassum S. Zhang Z. Y. Tan S. H. Long C. (2024). Urolithin A prevents sleep-deprivation-induced neuroinflammation and mitochondrial dysfunction in young and aged mice. Mol. Neurobiol. 61 (3), 1448–1466. 10.1007/s12035-023-03651-x 37725214

[B81] Morrison D. J. Preston T. (2016). Formation of short chain fatty acids by the gut microbiota and their impact on human metabolism. Gut Microbes 7 (3), 189–200. 10.1080/19490976.2015.1134082 26963409 PMC4939913

[B82] Muto H. Honda T. Tanaka T. Yokoyama S. Yamamoto K. Ito T. (2023). Proteomic analysis reveals changes in tight junctions in the small intestinal epithelium of mice fed a high-fat diet. Nutrients 15 (6), 1473. 10.3390/nu15061473 36986203 PMC10056729

[B83] Nian F. Chen Y. Xia Q. Zhu C. Wu L. Lu X. (2024). Gut microbiota metabolite trimethylamine N-oxide promoted NAFLD progression by exacerbating intestinal barrier disruption and intrahepatic cellular imbalance. Int. Immunopharmacol. 142 (Pt B), 113173. 10.1016/j.intimp.2024.113173 39298816

[B84] Nie Q. Li M. Huang C. Yuan Y. Liang Q. Ma X. (2024). The clinical efficacy and safety of berberine in the treatment of non-alcoholic fatty liver disease: a meta-analysis and systematic review. J. Transl. Med. 22 (1), 225. 10.1186/s12967-024-05011-2 38429794 PMC10908013

[B85] Niu Q. Li H. Tong L. Liu S. Zong W. Zhang S. (2023). TCMFP: a novel herbal formula prediction method based on network target's score integrated with semi-supervised learning genetic algorithms. Brief. Bioinform 24 (3), bbad102. 10.1093/bib/bbad102 36941113

[B86] Pan M. X. Zheng C. Y. Deng Y. J. Tang K. R. Nie H. Xie J. Q. (2021). Hepatic protective effects of Shenling Baizhu powder, a herbal compound, against inflammatory damage via TLR4/NLRP3 signalling pathway in rats with nonalcoholic fatty liver disease. J. Integr. Med. 19 (5), 428–438. 10.1016/j.joim.2021.07.004 34426178

[B87] Powell E. E. Wong V. W. Rinella M. (2021). Non-alcoholic fatty liver disease. Lancet 397 (10290), 2212–2224. 10.1016/S0140-6736(20)32511-3 33894145

[B88] Puri P. Daita K. Joyce A. Mirshahi F. Santhekadur P. K. Cazanave S. (2018). The presence and severity of nonalcoholic steatohepatitis is associated with specific changes in circulating bile acids. Hepatology 67 (2), 534–548. 10.1002/hep.29359 28696585 PMC5764808

[B89] Qian Y. Wang X. Cai L. Han J. Huang Z. Lou Y. (2024). Model informed precision medicine of Chinese herbal medicines formulas-A multi-scale mechanistic intelligent model. J. Pharm. Anal. 14 (4), 100914. 10.1016/j.jpha.2023.12.004 38694562 PMC11061219

[B90] Qu J. Tan S. Xie X. Wu W. Zhu H. Li H. (2021). Dendrobium officinale polysaccharide attenuates insulin resistance and abnormal lipid metabolism in obese mice. Front. Pharmacol. 12, 659626. 10.3389/fphar.2021.659626 34194325 PMC8236886

[B91] Rinella M. E. Lazarus J. V. Ratziu V. Francque S. M. Sanyal A. J. Kanwal F. (2023). A multisociety Delphi consensus statement on new fatty liver disease nomenclature. J. Hepatol. 79 (6), 1542–1556. 10.1016/j.jhep.2023.06.003 37364790

[B92] Rojas I. Y. Moyer B. J. Ringelberg C. S. Wilkins O. M. Pooler D. B. Ness D. B. (2021). Kynurenine-induced aryl hydrocarbon receptor signaling in mice causes body mass gain, liver steatosis, and hyperglycemia. Obes. (Silver Spring) 29 (2), 337–349. 10.1002/oby.23065 PMC10782555 33491319

[B93] Rong L. Zou J. Ran W. Qi X. Chen Y. Cui H. (2022). Advancements in the treatment of non-alcoholic fatty liver disease (NAFLD). Front. Endocrinol. (Lausanne) 13, 1087260. 10.3389/fendo.2022.1087260 36726464 PMC9884828

[B94] Saeed H. Diaz L. A. Gil-Gomez A. Burton J. Bajaj J. S. Romero-Gomez M. (2025). Microbiome-centered therapies for the management of metabolic dysfunction-associated steatotic liver disease. Clin. Mol. Hepatol. 31 (Suppl. l), S94–S111. 10.3350/cmh.2024.0811 39604327 PMC11925441

[B95] Safari Z. Gerard P. (2019). The links between the gut microbiome and non-alcoholic fatty liver disease (NAFLD). Cell Mol. Life Sci. 76 (8), 1541–1558. 10.1007/s00018-019-03011-w 30683985 PMC11105223

[B96] Sampson T. R. Debelius J. W. Thron T. Janssen S. Shastri G. G. Ilhan Z. E. (2016). Gut microbiota regulate motor deficits and neuroinflammation in a model of Parkinson's disease. Cell 167 (6), 1469–1480. 10.1016/j.cell.2016.11.018 27912057 PMC5718049

[B97] Sardinha-Silva A. Alves-Ferreira E. V. C. Grigg M. E. (2022). Intestinal immune responses to commensal and pathogenic protozoa. Front. Immunol. 13, 963723. 10.3389/fimmu.2022.963723 36211380 PMC9533738

[B98] Shi J. Liu Y. Zhang Z. Zhong X. Cao Y. Ni H. (2025). Zexie-Baizhu Decoction ameliorates non-alcoholic fatty liver disease through gut-adipose tissue crosstalk. J. Ethnopharmacol. 337 (Pt 1), 118700. 10.1016/j.jep.2024.118700 39182702

[B99] Shi L. Wang J. Wang Y. Feng Y. (2016). MDG-1, an Ophiopogon polysaccharide, alleviates hyperlipidemia in mice based on metabolic profile of bile acids. Carbohydr. Polym. 150, 74–81. 10.1016/j.carbpol.2016.05.008 27312615

[B100] Shu X. Li M. Cao Y. Li C. Zhou W. Ji G. (2021). Berberine alleviates non-alcoholic steatohepatitis through modulating gut microbiota mediated intestinal FXR activation. Front. Pharmacol. 12, 750826. 10.3389/fphar.2021.750826 34603061 PMC8484326

[B101] Si X. Shang W. Zhou Z. Shui G. Lam S. M. Blanchard C. (2018). Gamma-aminobutyric acid enriched rice bran diet attenuates insulin resistance and balances energy expenditure via modification of gut microbiota and short-chain fatty acids. J. Agric. Food Chem. 66 (4), 881–890. 10.1021/acs.jafc.7b04994 29327584

[B102] Singh T. P. Kadyan S. Devi H. Park G. Nagpal R. (2023). Gut microbiome as a therapeutic target for liver diseases. Life Sci. 322, 121685. 10.1016/j.lfs.2023.121685 37044173

[B145] Sun L. Xie C. Wang G. Wu Y. Wu Q. Wang X. (2018). Gut microbiota and intestinal FXR mediate the clinical benefits of metformin. Nat. Med 24 (12), 1919–1929. 10.1038/s41591-018-0222-4 30397356 PMC6479226

[B103] Tahara A. (2021). Effects of SGLT2 inhibitor ipragliflozin alone and combined with pioglitazone on fluid retention in type 2 diabetic mice with NASH. Eur. J. Pharmacol. 901, 174076. 10.1016/j.ejphar.2021.174076 33798599

[B104] Tan X. Liu Y. Long J. Chen S. Liao G. Wu S. (2019). Trimethylamine N-oxide aggravates liver steatosis through modulation of bile acid metabolism and inhibition of farnesoid X receptor signaling in nonalcoholic fatty liver disease. Mol. Nutr. Food Res. 63 (17), e1900257. 10.1002/mnfr.201900257 31095863

[B105] Targher G. Tilg H. Byrne C. D. (2021). Non-alcoholic fatty liver disease: a multisystem disease requiring a multidisciplinary and holistic approach. Lancet Gastroenterol. Hepatol. 6 (7), 578–588. 10.1016/S2468-1253(21)00020-0 33961787

[B106] Teng T. Qiu S. Zhao Y. Zhao S. Sun D. Hou L. (2022). Pathogenesis and therapeutic strategies related to non-alcoholic fatty liver disease. Int. J. Mol. Sci. 23 (14), 7841. 10.3390/ijms23147841 35887189 PMC9322253

[B107] Tian G. Wang W. Xia E. Chen W. Zhang S. (2023). Dendrobium officinale alleviates high-fat diet-induced nonalcoholic steatohepatitis by modulating gut microbiota. Front. Cell Infect. Microbiol. 13, 1078447. 10.3389/fcimb.2023.1078447 36860985 PMC9968977

[B108] Tilg H. Adolph T. E. Trauner M. (2022). Gut-liver axis: pathophysiological concepts and clinical implications. Cell Metab. 34 (11), 1700–1718. 10.1016/j.cmet.2022.09.017 36208625

[B109] Ueno M. Fujita Y. Tanaka T. Nakamura Y. Kikuta J. Ishii M. (2013). Layer V cortical neurons require microglial support for survival during postnatal development. Nat. Neurosci. 16 (5), 543–551. 10.1038/nn.3358 23525041

[B110] Unamuno X. Gomez-Ambrosi J. Ramirez B. Rodriguez A. Becerril S. Valenti V. (2021). NLRP3 inflammasome blockade reduces adipose tissue inflammation and extracellular matrix remodeling. Cell Mol. Immunol. 18 (4), 1045–1057. 10.1038/s41423-019-0296-z 31551515 PMC8115140

[B111] Vacca M. Kamzolas I. Harder L. M. Oakley F. Trautwein C. Hatting M. (2024). An unbiased ranking of murine dietary models based on their proximity to human metabolic dysfunction-associated steatotic liver disease (MASLD). Nat. Metab. 6 (6), 1178–1196. 10.1038/s42255-024-01043-6 38867022 PMC11199145

[B112] Wang G. Huang W. Xia Y. Xiong Z. Ai L. (2019a). Cholesterol-lowering potentials of Lactobacillus strain overexpression of bile salt hydrolase on high cholesterol diet-induced hypercholesterolemic mice. Food Funct. 10 (3), 1684–1695. 10.1039/c8fo02181c 30839966

[B113] Wang J. Zang J. Yu Y. Liu Y. Cao H. Guo R. (2024a). Lingguizhugan oral solution alleviates MASLD by regulating bile acids metabolism and the gut microbiota through activating FXR/TGR5 signaling pathways. Front. Pharmacol. 15, 1426049. 10.3389/fphar.2024.1426049 39211777 PMC11358101

[B114] Wang L. Li W. Li Y. Chen G. Zhao L. Li W. (2024b). Dried tangerine peel polysaccharide (DTPP) alleviates hepatic steatosis by suppressing TLR4/MD-2-mediated inflammation and endoplasmic reticulum stress. Bioorg Chem. 147, 107369. 10.1016/j.bioorg.2024.107369 38640721

[B115] Wang X. Shi L. Joyce S. Wang Y. Feng Y. (2017). MDG-1, a potential regulator of PPARα and PPARγ, ameliorates dyslipidemia in mice. Int. J. Mol. Sci. 18 (9), 1930. 10.3390/ijms18091930 28885549 PMC5618579

[B116] Wang X. Shi L. Wang X. Feng Y. Wang Y. (2019b). MDG-1, an Ophiopogon polysaccharide, restrains process of non-alcoholic fatty liver disease via modulating the gut-liver axis. Int. J. Biol. Macromol. 141, 1013–1021. 10.1016/j.ijbiomac.2019.09.007 31491513

[B117] Wells J. M. Brummer R. J. Derrien M. MacDonald T. T. Troost F. Cani P. D. (2017). Homeostasis of the gut barrier and potential biomarkers. Am. J. Physiol. Gastrointest. Liver Physiol. 312 (3), G171–G193. 10.1152/ajpgi.00048.2015 27908847 PMC5440615

[B118] Wu H. Lou T. Pan M. Wei Z. Yang X. Liu L. (2024). Chaihu Guizhi Ganjiang Decoction attenuates nonalcoholic steatohepatitis by enhancing intestinal barrier integrity and ameliorating PPARα mediated lipotoxicity. J. Ethnopharmacol. 326, 117841. 10.1016/j.jep.2024.117841 38310988

[B119] Wu L. Feng J. Li J. Yu Q. Ji J. Wu J. (2021). The gut microbiome-bile acid axis in hepatocarcinogenesis. Biomed. Pharmacother. 133, 111036. 10.1016/j.biopha.2020.111036 33378947

[B120] Xiang H. Sun D. Liu X. She Z. G. Chen Y. (2022). The role of the intestinal microbiota in nonalcoholic steatohepatitis. Front. Endocrinol. (Lausanne) 13, 812610. 10.3389/fendo.2022.812610 35211093 PMC8861316

[B121] Xu D. X. Guo X. X. Zeng Z. Wang Y. Qiu J. (2021). Puerarin improves hepatic glucose and lipid homeostasis *in vitro* and *in vivo* by regulating the AMPK pathway. Food Funct. 12 (6), 2726–2740. 10.1039/d0fo02761h 33681875

[B122] Xue L. Deng Z. Luo W. He X. Chen Y. (2022). Effect of fecal microbiota transplantation on non-alcoholic fatty liver disease: a randomized clinical trial. Front. Cell Infect. Microbiol. 12, 759306. 10.3389/fcimb.2022.759306 35860380 PMC9289257

[B123] Yamada S. Takashina Y. Watanabe M. Nagamine R. Saito Y. Kamada N. (2018). Bile acid metabolism regulated by the gut microbiota promotes non-alcoholic steatohepatitis-associated hepatocellular carcinoma in mice. Oncotarget 9 (11), 9925–9939. 10.18632/oncotarget.24066 29515780 PMC5839411

[B124] Yan H. M. Xia M. F. Wang Y. Chang X. X. Yao X. Z. Rao S. X. (2015). Efficacy of berberine in patients with non-alcoholic fatty liver disease. PLoS One 10 (8), e0134172. 10.1371/journal.pone.0134172 26252777 PMC4529214

[B125] Yang J. Chen H. Nie Q. Huang X. Nie S. (2020). Dendrobium officinale polysaccharide ameliorates the liver metabolism disorders of type II diabetic rats. Int. J. Biol. Macromol. 164, 1939–1948. 10.1016/j.ijbiomac.2020.08.007 32763406

[B126] Yang Y. Li M. Wang Q. Huang H. Zhao Y. Du F. (2022). Pueraria lobata starch regulates gut microbiota and alleviates high-fat high-cholesterol diet induced non-alcoholic fatty liver disease in mice. Food Res. Int. 157, 111401. 10.1016/j.foodres.2022.111401 35761655

[B127] Yao Z. Guo J. Du B. Hong L. Zhu Y. Feng X. (2023). Effects of Shenling Baizhu powder on intestinal microflora metabolites and liver mitochondrial energy metabolism in nonalcoholic fatty liver mice. Front. Microbiol. 14, 1147067. 10.3389/fmicb.2023.1147067 37538846 PMC10394096

[B128] Yu S. Jiang J. Li Q. Liu X. Wang Z. Yang L. (2022). Schisantherin A alleviates non-alcoholic fatty liver disease by restoring intestinal barrier function. Front. Cell Infect. Microbiol. 12, 855008. 10.3389/fcimb.2022.855008 36132991 PMC9483129

[B129] Yuan J. Chen C. Cui J. Lu J. Yan C. Wei X. (2019). Fatty liver disease caused by high-alcohol-producing *Klebsiella pneumoniae* . Cell Metab. 30 (6), 675–688. 10.1016/j.cmet.2019.08.018 31543403

[B130] Zaufel A. van de Wiel S. M. W. Yin L. Fauler G. Chien D. Dong X. (2021). Secondary (iso)BAs cooperate with endogenous ligands to activate FXR under physiological and pathological conditions. Biochim. Biophys. Acta Mol. Basis Dis. 1867 (8), 166153. 10.1016/j.bbadis.2021.166153 33895309 PMC8177068

[B131] Zhai Y. Zhou W. Yan X. Qiao Y. Guan L. Zhang Z. (2022). Astragaloside IV ameliorates diet-induced hepatic steatosis in obese mice by inhibiting intestinal FXR via intestinal flora remodeling. Phytomedicine 107, 154444. 10.1016/j.phymed.2022.154444 36155217

[B132] Zhang F. Zhao S. Yan W. Xia Y. Chen X. Wang W. (2016). Branched chain amino acids cause liver injury in obese/diabetic mice by promoting adipocyte lipolysis and inhibiting hepatic autophagy. EBioMedicine 13, 157–167. 10.1016/j.ebiom.2016.10.013 27843095 PMC5264279

[B133] Zhang Y. Tang K. Deng Y. Chen R. Liang S. Xie H. (2018). Effects of shenling baizhu powder herbal formula on intestinal microbiota in high-fat diet-induced NAFLD rats. Biomed. Pharmacother. 102, 1025–1036. 10.1016/j.biopha.2018.03.158 29710519

[B134] Zhao Z. H. Wang Z. X. Zhou D. Han Y. Ma F. Hu Z. (2021). Sodium butyrate supplementation inhibits hepatic steatosis by stimulating liver kinase B1 and insulin-induced gene. Cell Mol. Gastroenterol. Hepatol. 12 (3), 857–871. 10.1016/j.jcmgh.2021.05.006 33989817 PMC8346675

[B135] Zhao Z. H. Xin F. Z. Xue Y. Hu Z. Han Y. Ma F. (2019). Indole-3-propionic acid inhibits gut dysbiosis and endotoxin leakage to attenuate steatohepatitis in rats. Exp. Mol. Med. 51 (9), 1–14. 10.1038/s12276-019-0304-5 PMC6802644 31506421

[B136] Zheng N. Wang H. Zhu W. Li Y. Li H. (2024). Astragalus polysaccharide attenuates nonalcoholic fatty liver disease through THDCA in high-fat diet-fed mice. J. Ethnopharmacol. 320, 117401. 10.1016/j.jep.2023.117401 37967775

[B137] Zhong M. Yan Y. Yuan H. A R. Xu G. Cai F. (2022). Astragalus mongholicus polysaccharides ameliorate hepatic lipid accumulation and inflammation as well as modulate gut microbiota in NAFLD rats. Food Funct. 13 (13), 7287–7301. 10.1039/d2fo01009g 35726797

[B138] Zhong X. C. Liu Y. M. Gao X. X. Krausz K. W. Niu B. Gonzalez F. J. (2023). Caffeic acid phenethyl ester suppresses intestinal FXR signaling and ameliorates nonalcoholic fatty liver disease by inhibiting bacterial bile salt hydrolase activity. Acta Pharmacol. Sin. 44 (1), 145–156. 10.1038/s41401-022-00921-7 35655096 PMC9813015

[B139] Zhou J. Zhang N. Aldhahrani A. Soliman M. M. Zhang L. Zhou F. (2022). Puerarin ameliorates nonalcoholic fatty liver in rats by regulating hepatic lipid accumulation, oxidative stress, and inflammation. Front. Immunol. 13, 956688. 10.3389/fimmu.2022.956688 35958617 PMC9359096

[B140] Zhou R. Wang Y. Chen S. Cheng F. Yi Y. Lv C. (2025). Anti-inflammatory effect of *Dendrobium officinale* extract on high-fat diet-induced obesity in rats: involvement of gut microbiota, liver transcriptomics, and NF-κB/IκB pathway. Antioxidants (Basel) 14 (4), 432. 10.3390/antiox14040432 40298780 PMC12024317

[B141] Zhou Y. X. Zhang H. Peng C. (2014). Puerarin: a review of pharmacological effects. Phytother. Res. 28 (7), 961–975. 10.1002/ptr.5083 24339367

[B142] Zhu L. Baker S. S. Gill C. Liu W. Alkhouri R. Baker R. D. (2013). Characterization of gut microbiomes in nonalcoholic steatohepatitis (NASH) patients: a connection between endogenous alcohol and NASH. Hepatology 57 (2), 601–609. 10.1002/hep.26093 23055155

[B143] Zhu Y. L. Meng L. L. Ma J. H. Yuan X. Chen S. W. Yi X. R. (2024). Loss of *LBP* triggers lipid metabolic disorder through H3K27 acetylation-mediated C/EBPβ- *SCD* activation in non-alcoholic fatty liver disease. Zool. Res. 45 (1), 79–94. 10.24272/j.issn.2095-8137.2023.022 38114435 PMC10839665

[B144] Zou J. Xiang Q. Tan D. Shi L. Liu X. Wu Y. (2023). Zuogui-Jiangtang-Qinggan-Fang alleviates high-fat diet-induced type 2 diabetes mellitus with non-alcoholic fatty liver disease by modulating gut microbiome-metabolites-short chain fatty acid composition. Biomed. Pharmacother. 157, 114002. 10.1016/j.biopha.2022.114002 36410120

